# A functional screen for copper homeostasis genes identifies a pharmacologically tractable cellular system

**DOI:** 10.1186/1471-2164-15-263

**Published:** 2014-04-05

**Authors:** Ulrich Schlecht, Sundari Suresh, Weihong Xu, Ana Maria Aparicio, Angela Chu, Michael J Proctor, Ronald W Davis, Curt Scharfe, Robert P St Onge

**Affiliations:** 1 Stanford Genome Technology Center, Department of Biochemistry, Stanford University, 855 S California Avenue, Palo Alto, CA 94304, USA

**Keywords:** Copper, Iron, Yeast, Functional Genomics, Disulfiram, Elesclomol, Menkes, Wilson’s, Vacuole

## Abstract

**Background:**

Copper is essential for the survival of aerobic organisms. If copper is not properly regulated in the body however, it can be extremely cytotoxic and genetic mutations that compromise copper homeostasis result in severe clinical phenotypes. Understanding how cells maintain optimal copper levels is therefore highly relevant to human health.

**Results:**

We found that addition of copper (Cu) to culture medium leads to increased respiratory growth of yeast, a phenotype which we then systematically and quantitatively measured in 5050 homozygous diploid deletion strains. Cu’s positive effect on respiratory growth was quantitatively reduced in deletion strains representing 73 different genes, the function of which identify increased iron uptake as a cause of the increase in growth rate. Conversely, these effects were enhanced in strains representing 93 genes. Many of these strains exhibited respiratory defects that were specifically rescued by supplementing the growth medium with Cu. Among the genes identified are known and direct regulators of copper homeostasis, genes required to maintain low vacuolar pH, and genes where evidence supporting a functional link with Cu has been heretofore lacking. Roughly half of the genes are conserved in man, and several of these are associated with Mendelian disorders, including the Cu-imbalance syndromes Menkes and Wilson’s disease. We additionally demonstrate that pharmacological agents, including the approved drug disulfiram, can rescue Cu-deficiencies of both environmental and genetic origin.

**Conclusions:**

A functional screen in yeast has expanded the list of genes required for Cu-dependent fitness, revealing a complex cellular system with implications for human health. Respiratory fitness defects arising from perturbations in this system can be corrected with pharmacological agents that increase intracellular copper concentrations.

## Background

Copper is an essential element for most living organisms. Its incorporation into specific enzymes is required for catalysis of several vital, and highly conserved cellular processes [1]. For example, copper binding to specific sites in cytochrome c oxidase (*i.e.* complex IV of the electron transport chain) is required for complex assembly and stability, electron transfer activity, and ultimately respiratory metabolism [2]. Copper metallation also has important roles in responding to oxidative stress, by activating superoxide dismutase (SOD1) [3,4], and in regulating iron import by activating ferroxidases, which oxidize substrates for iron transporters [5-7].

The unique redox chemistry of copper ions, which allows it to exist in an oxidized (Cu2+) or reduced (Cu1+) state, is key to its catalytic properties but also contributes to its production of reactive oxygen species (ROS). To keep this deleterious capacity in check, the cell has evolved mechanisms to maintain tight regulatory control of copper storage and transport [1]. Metallochaperones protect the cell from copper toxicity by binding to free copper and facilitating its transport to, and incorporation into, important target proteins [8]. The yeast cytosolic chaperone Atx1 for example, delivers copper to the Ccc2 ATPase, which transports copper into the Golgi where it activates the Fet3 ferrooxidase, ultimately leading to iron uptake.

The importance of maintaining proper copper homeostasis is underscored by several rare, yet severe, genetic diseases. Wilson’s disease is an autosomal recessive disorder resulting from mutations in the copper transporter ATP7B, the human ortholog of yeast *CCC2*. Patients express a variety of neurological, psychiatric, and hepatic problems arising from abnormal copper accumulation, primarily in the brain and liver [9]. Mutations in the related gene ATP7A cause the X-linked recessive disorder Menkes disease [10]. Notably, the clinical symptoms of this disease arise from copper deficiency, and include neurological defects (mental retardation, seizures), growth retardation, hypothermia and “kinky” or “steely” hair [11]. Mitochondrial complex IV disorders, including Leigh syndrome, have also been linked to genes with important roles in copper metabolism, including SCO1, SCO2, and SURF-1 [12-19]. Abnormal copper concentrations have also been observed in Alzheimer’s and Huntington’s disease [20-22], and copper has been shown to promote aggregation of the α-synuclein protein, a hallmark of Parkinson’s disease [23].

Yeast has been an exceptionally useful model organism for deciphering the cellular mechanisms regulating copper homeostasis [24,25], and indeed yeast orthologs exist for most of the disease genes described above. Here, we leverage the dependency between respiratory growth rate and copper availability to conduct a systematic screen for genes associated with copper homeostasis. This screen identified genes with known roles in copper regulation, and those with no previously identified functional association with copper. Consistent with the vacuole being a cellular store for copper, many of the genes identified are required for vacuole acidification, and we go on to show that copper is in fact limiting under conditions of vacuole stress. Several genes were also orthologs of human disease genes that have not been previously linked to copper imbalance. Finally, we demonstrate that the approved drug disulfiram (DSF), and the experimental drug elesclomol (ES), can rescue respiratory growth defects arising from copper deficiency.

## Results and discussion

### Respiratory fitness and copper

Though yeast preferentially ferment glucose for energy, they are able to switch to oxidative phosphorylation in the absence of a fermentable carbon source [26,27]. This attribute, in addition to its simple growth requirements, makes yeast a powerful model system for identifying chemical probes targeting energy metabolism [28]. To identify chemical compounds that ‘boost’ respiratory growth, and potentially serve as leads for human mitochondrial diseases, we conducted a phenotypic screen of ~3000 natural product derivatives (TimTec) for compounds that accelerated growth of respiring yeast (grown in Yeast Extract, Peptone, Ethanol, Glycerol medium; YPEG), but not fermenting yeast (grown in Yeast Extract, Peptone, Dextrose medium; YPD). This screen identified a series of structurally related compounds with the desired effect, but we determined that this effect was attributed to the CuCl_2_ salt with which each compound was supplied (Additional file 1: Figure S1A and S1B).

**Figure 1:**
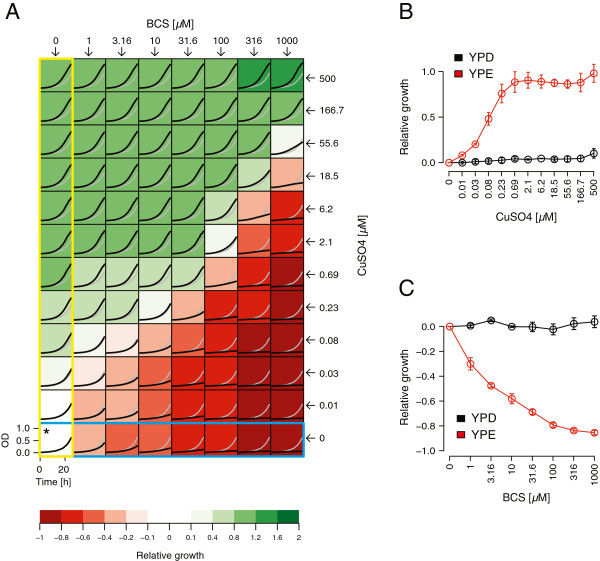
**Media copper concentrations influence respiratory, but not fermentative, growth in yeast. (A)** Dose–response matrix measuring the combinatorial effects of copper sulfate (CuSO_4_) and bathocuproine disulphonic acid (BCS, a copper chelator) on respiratory growth. Yeast (BY4743) was grown under respiring conditions (YPE media) in increasing concentrations of CuSO_4_ (see right) and BCS (see top). Optical density (*i.e.* OD_600_, y-axis) of each culture is plotted over time (x-axis). Each condition is colored based on growth relative to the untreated condition (*i.e.* no BCS and no CuSO_4_ added; marked with an asterisk and represented by the grey curve). Green and red indicate increased and decreased growth relative to this reference, respectively (see legend below). Growth data highlighted in yellow and blue were used in **(B)** and **(C)**, respectively. **(B)** Dose–response curves illustrating the effect of CuSO_4_ (x-axis) on yeast growth. Growth relative to that in the absence of CuSO_4_ is plotted on the y-axis, and was measured in both fermenting (YPD) and respiring (YPE) conditions (indicated in black and red, respectively). The mean of three replicates is plotted; error bars represent the standard deviation. **(C)** Similar to **(B)** but measuring the effects of BCS (x-axis).

To further examine the effect of copper ions on respiratory growth, yeast (BY4743) was grown in YP media containing the non-fermentable carbon source ethanol (YPE), at various concentrations of copper sulfate (CuSO_4_). Growth of 100 μl cultures was monitored in 96well plates by measuring the increase in absorbance over time (see Methods). Supplemental copper concentrations as low as 10 nM were found to boost growth (Figure 1A and B). This boost was dose-dependent, but plateaued at 2 μM, indicating a saturation of copper above this concentration. In contrast, no such boost was observed in dextrose-containing media (YPD) (Figure 1B). This positive effect of Cu was further confirmed to be specific to respiratory growth conditions by comparing growth in other fermentable (galactose) and non-fermentable (glycerol and lactate) carbon sources (Additional file 1: Figure S1C). Similar results were obtained using spot assays on agar plates. When respiring yeast were pre-grown in the presence of copper-supplemented media, no significant increase in the number of colony forming units was observed, however, the size of each colony was substantially increased (Additional file 1: Figure S1D). From this we conclude that copper (at the concentration tested) does not dramatically influence yeast viability, but rather, increases growth under respiratory conditions.

We also tested the effect of copper depletion on respiratory growth, by culturing yeast in increasing concentrations (1 – 1000 μM) of the copper chelator, bathocuproine disulphonic acid (BCS). A significant dose-dependent reduction in growth was observed at BCS concentrations as low as 1 μM, and growth was strongly inhibited at BCS concentrations higher than 100 μM (Figure 1A and C). This inhibitory effect, however, was attenuated by addition of CuSO_4_ to the growth medium (Figure 1A). For example, the addition of 500 μM CuSO_4_ completely suppressed the inhibitory effects of BCS. Similar experiments using YPD media showed that addition of BCS does not alter fermentative growth (Figure 1C). Collectively, these results demonstrate a critical dependency between respiratory growth and copper ion concentrations in the growth medium.

### A screen for copper homeostasis genes

To better understand the underlying cellular mechanisms controlling copper-dependent respiratory growth, we used the yeast homozygous gene deletion collection, in which individual diploid strains have both copies of a non-essential gene deleted. This collection has been a valuable resource for genome-scale analysis of conditions affecting growth [29-31]. Each strain contains unique 20-bp “barcode” sequences flanked by common priming sites, allowing the relative abundance of individual strains to be quantitatively monitored within a pool of competitively-grown strains [32,33]. Previous work has used this resource to identify genes that protect fermenting yeast from toxic concentrations of copper [34]. To identify genes that are important for Cu-dependent respiratory growth, we measured the fitness of 5050 homozygous deletion strains (Additional file 2: Table S1) in parallel in rich media (*i.e.* Yeast Extract, Peptone) using dextrose, ethanol, glycerol, or lactate as a carbon source, in the presence or absence of 500 μM CuSO_4_ (a concentration that increases respiratory growth rate of the parental strain). Each condition was tested in triplicate. CEL files are available via the NCBI's Gene Expression Omnibus [35] under accession number GSE47175. Though it was not a goal of this study, examination of this dataset reveals genes that are specifically required for metabolizing different non-fermentable carbon sources. For example, the *fsh1Δ/fsh1Δ* deletion strain showed an ethanol-specific growth defect (Additional file 1: Figure S2). This suggests a role for *FSH1*, a putative serine hydrolase that has sequence-similarity to the human candidate tumor suppressor OVCA2 [36,37], in ethanol metabolism.

Importantly, these experiments identified a group of 313 respiratory-deficient strains (see Methods and Additional file 2: Table S1) that grew in YPD but were undetectable following competitive growth in media using non-fermentable carbon sources. As expected, the majority (259; ~83%) of these strains were previously identified in a screen for mutants with impaired mitochondrial respiration [31]. Genes deleted in these strains were also enriched for the biological processes *mitochondrion organization* (*p-value*: 1.44e-99) and *cellular respiration* (*p-value*: 2.03e-22). We reasoned that the inability of these strains to compete with respiratory-proficient strains in our competitive assay may preclude accurate measurement of their response to Cu. Therefore, to better assay these strains, we assembled a smaller pool consisting only of these 313 strains, plus an additional 18 strains of interest (see Material and Methods), and interrogated this pool in YPE, in the presence or absence of 500 μM CuSO_4_.

**Figure 2:**
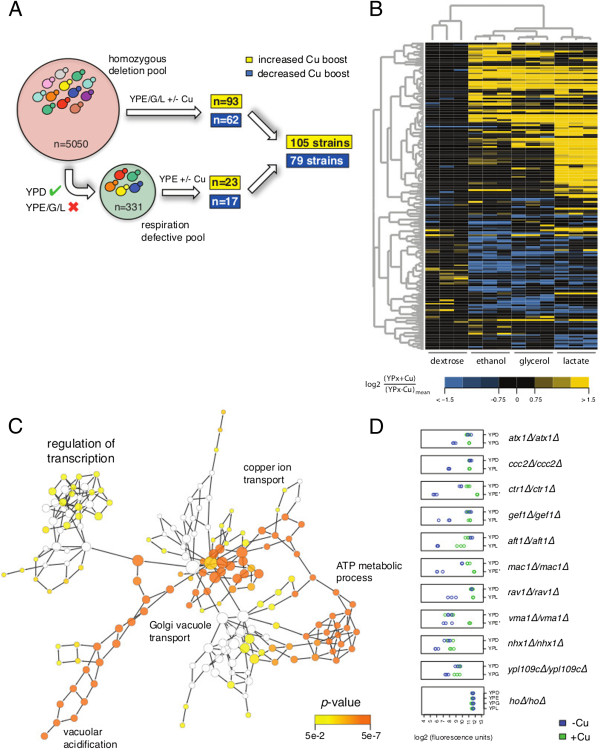
**A systematic screen for genes involved in Cu-dependent growth. (A)** Schematic of the experimental workflow. A complete pool of homozygous deletion mutants and a smaller pool of respiration deficient strains (see Methods) were grown under respiratory conditions in the presence and absence of CuSO_4_. Comparison of CuSO_4_-treated and -untreated samples, identified 105 and 79 strains where Cu’s effect on growth was increased or decreased, respectively (see Methods). **(B)** Heatmap depicting log2 ratios of CuSO_4_-treated and -untreated samples (see legend below) for the 184 strains identified in **(A)**. Yellow indicates greater Cu-dependent growth and blue indicates reduced Cu-dependent growth. Dendrograms depict the results from hierarchical clustering of genes (y-axis) and experiments (x-axis). **(C)** Network illustrating overrepresented Gene Ontology categories (nodes) among the 105 strains with an enhanced response to copper. This gene list was analyzed, and the network image created, using the BiNGO plugin for Cytoscape [38] (see Methods). Representative nodes are circled and labelled with those genes identified in the screen. **(D)** Dotplots of log2-transformed microarray fluorescence intensities for a select set of deletion strains. Triplicate data points for YPD and the non-fermentable carbon source in which the greatest copper response was observed are shown for copper-treated (green) and -untreated (blue) conditions. YPE, YPG, and YPL refer to data from the complete homozygous deletion pool experiment; YPE* refers to the respiration defective pool.

The effect of CuSO_4_ on the collective growth of the pools was similar to that observed for BY4743 in Figure 1. To identify individual strains where this effect was altered, we compared the abundance of each barcode in the copper-treated samples to untreated samples, for both deletion pools, and for each non-fermentable carbon source (see Figure 2A). Using a stringent cutoff (log2 fold change > 1.5 and q-value < 0.05; see Methods), we identified 79 deletion strains in which Cu’s positive effect on respiratory growth was quantitatively reduced, and 105 strains in which Cu’s positive effects were substantively greater (compared to other strains in the pool) in at least one non-fermentable carbon source (Additional file 2: Table S1 and Additional file 1: Figure S3 and S4). The latter set was largely comprised of strains with respiratory growth defects that were specifically rescued by the addition of CuSO_4_. However, the majority of strains with respiratory growth defects were not substantively aided by the addition of CuSO_4_ (Additional file 1: Figure S5), underscoring that the copper-rescue effect was specific to the 105 strains identified.

We applied hierarchical clustering to the Cu-induced fitness changes observed in each of the four carbon sources for these 184 strains (Figure 2B). An examination of the clusters across the experiment axis showed that all replicate experiments correlated as nearest neighbors, confirming that the genome-wide data were highly reproducible. Samples grown in YPD clustered separately from the non-fermentable (YPE, YPG, and YPL) carbon-sources and in YPD, the fitness of most of these strains was not greatly affected by the addition of CuSO_4_. This is consistent with copper affecting respiratory, but not fermentative growth at the concentration tested. While the results were generally consistent between the non-fermentable carbon sources, carbon source-specific effects were apparent as well. Most notably, growth in lactate identified many more strains exhibiting an increased Cu boost than did growth in ethanol or glycerol. This difference may be attributed to the higher respiratory fitness of many strains in YPL (due to the lower pH of this media; discussed further below) which facilitates their detection in our competitive assay. While this explanation attributes carbon-source specific results to technical limitations of the competitive growth assay (*i.e.* slow growing strains dropping out of the pool in YPE and YPG, but not YPL), it is also possible that mechanistic differences in the catabolism of ethanol, glycerol, and lactate result in discrete genetic dependencies for the copper response. Further experimentation will be needed to determine whether such cases do indeed exist.

To search for functional enrichment among the genes that were deleted in the 105 and 79 strains identified above, we used the Biological Networks Gene Ontology plugin (BiNGO) for Cytoscape (see Methods). The two gene lists contained 11 and 6 ‘dubious’ open-reading frames, respectively, that partially overlapped with a verified gene. In several instances these strains confirmed the results for verified genes (such as the dubious ORFs *YDR203W* and *YDR455C* which overlap with *RAV2* and *NHX1*, respectively). One gene, *ADA2,* was represented twice in the deletion pool and both strains were identified in our screen. For our Gene Ontology (GO) analysis we used the non-redundant set of genes. The 73 genes that were required for Cu-dependent growth (*i.e.* whose deletion diminished the Cu-dependent growth boost), were significantly enriched for several GO categories, including *2-oxoglutarate metabolic process*, and *iron ion homeostasis* (Additional file 1: Figure S6 and Additional file 2: Table S2). Among the genes belonging to the latter term is *FET3*, which encodes the high-affinity iron uptake protein that links iron and copper homeostasis [39]. The dependence of copper’s effects on Fet3 was verified in isogenic cultures, and in fact, the *fet3Δ*/*fet3Δ* strain exhibited reduced (not enhanced) growth in 500 μM CuSO_4_ (Additional file 1: Figure S7A). Copper-limiting conditions are known to result in cellular iron deficiency [24,40], and we found that addition of high concentrations of FeSO_4_ was itself sufficient to enhance respiratory growth of yeast (Additional file 1: Figure S7B). Therefore, to further explore the role of iron, we repeated the experiment described in Figure 1A in the presence of excess FeSO_4_. Addition of 1 mM FeSO_4_ was found to confer resistance to low concentrations (≤10 μM) of BCS, and to effectively mask the growth advantages imparted by CuSO_4_ under these conditions (Additional file 1: Figure S7C). Collectively, these data suggest that increased iron uptake contributes to the respiratory growth increase arising from copper supplementation.

In contrast, the 93 genes in the enhanced growth group were significantly enriched for the GO processes *Golgi to vacuole transport* and *vacuolar acidification* (Figure 2C, Additional file 1: Figure S6 and Additional file 2: Table S3). Among this list of 93 genes are three genes that are directly linked to copper transport in yeast (*CTR1, ATX1* and *CCC2*) and in higher eukaryotes (*hCTR1/2*, *Atox1* and *Atp7A/B*). Deletion of these genes was previously shown to result in growth defects on low copper media [25]. The cytosolic copper metallochaperone Atx1 transports Cu ions from the high-affinity copper transporter Ctr1 in the plasma membrane to Ccc2 on post-Golgi vesicles to eventually target it to Fet3 on the cell surface [6,7,41,42]. Our screen also identified *GEF1*, a chloride channel involved in cation homeostasis. The Gef1 protein co-localizes with Ccc2 at late- and post-Golgi vesicles and is required for loading Cu ions onto Fet3 [43]. For other genes identified by our screen, evidence supporting a functional role in cellular copper homeostasis is either limited or lacking entirely. These include *YPL109C*, which encodes a protein of unknown function that was detected in highly purified mitochondria [44,45]. Figure 2D shows quantile-normalized fluorescence values for several of the deletion strains specifically discussed herein. The complete results for all strains identified in our screen are shown in Additional file 1: Figure S3 and S4. Collectively, these data will be a rich source of information for future characterization of genes involved in copper homeostasis.

Previous work in yeast has demonstrated that mitochondria maintain a non-proteinaceous copper pool that exceeds CcO-associated copper concentrations [46]. It is thought that this pool may serve as a source of copper ions for chaperones that mediate metallation of CcO, which occurs at two CcO subunits encoded by the mitochondrial genome, Cox1 and Cox2 [8]. The metallochaperone Cox17 transfers copper to two mitochondrial inner membrane proteins Cox11 and Sco1 [47], which then mediate metallation of Cox1 and Cox2, respectively [8]. It is notable that our genomic screen did not identify the *cox17*, *cox11*, or *sco1* deletion strains as being rescued by copper. These results are consistent with previous analysis of *sco1* null strains [48], and of *cox17* null strains, whose respiratory growth defect was rescued by copper, but only at concentrations much higher than those used in the present study [49].

### Vacuole acidity and copper homeostasis

The yeast vacuole, generally regarded as the functional equivalent of the mammalian lysosome, is an acidic organelle with a variety of important functions including: protein degradation, ion and small-molecule storage, and pH homeostasis [50,51]. Consistent with previous work demonstrating an important role for the vacuole in storage and mobilization of Cu ions [1,52-54], several genes that maintain vacuolar pH, when deleted, were found to produce respiratory growth defects that were rescued by copper. These included numerous subunits of the vacuolar H^+^−ATPase (such as *VMA1, VMA2, VMA3, VMA4, VMA6, VMA7, VMA11, VMA13, VMA16,* and *STV1*) and 2 subunits of the RAVE complex (*RAV1* and *RAV2*) which promotes assembly of the V-ATPase holoenzyme. Mobilization of vacuolar copper is thought to involve the Ctr2 protein, a copper transporter that localizes to the vacuolar membranes of budding yeast [52,53], fission yeast [55], and mammalian cells [56]. Though a *ctr2Δ* mutant was not included in our deletion collection and thus not identified in our screen, we did identify the *CTR2*-regulating transcription factors *AFT1* and *MAC1*[57].

**Figure 3:**
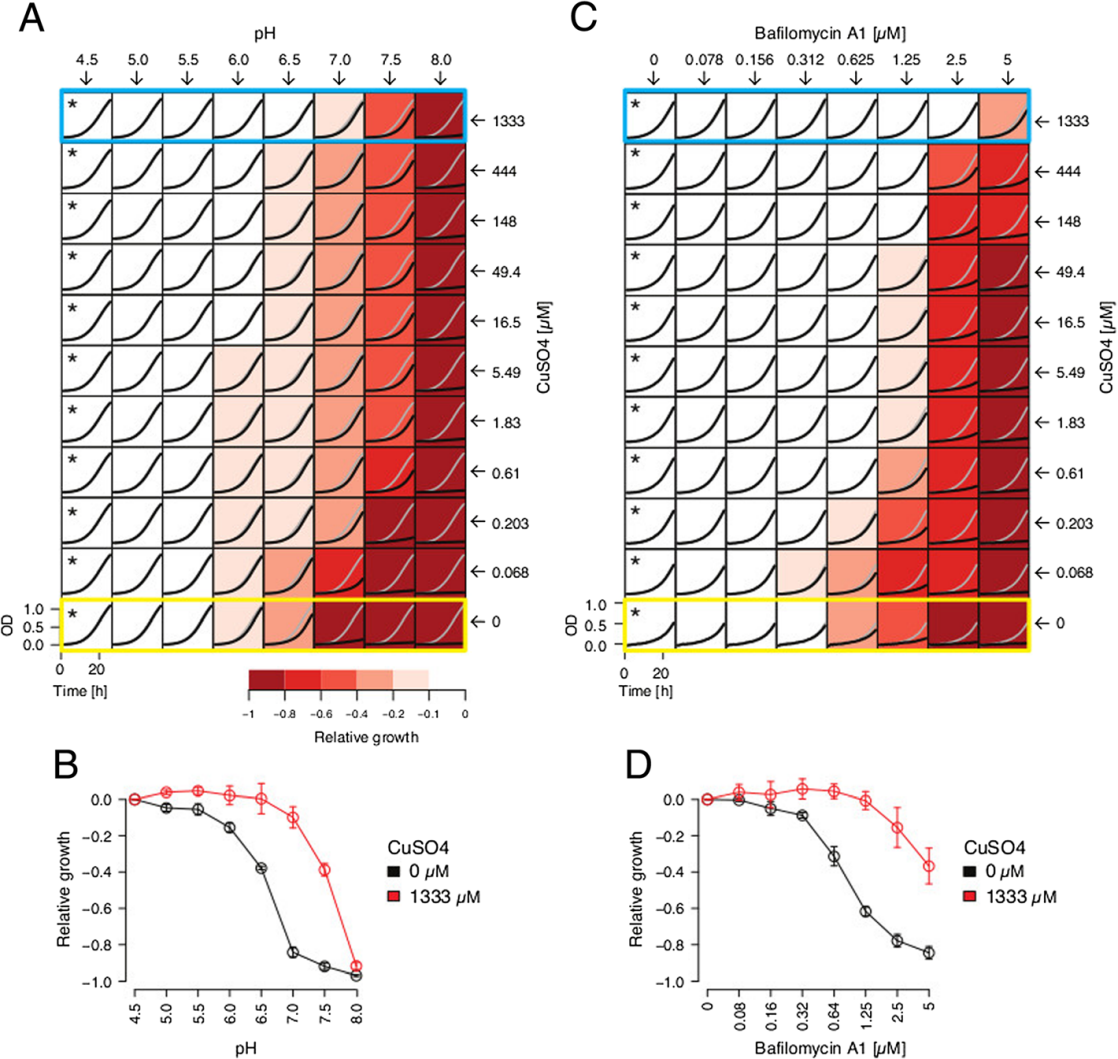
**Vacuolar pH plays a critical role in maintaining copper homeostasis. (A)** Dose–response matrix measuring the combinatorial effects of CuSO_4_ and media pH on respiratory growth. BY4743 was grown in buffered YPE media of increasing pH (see top), in multiple concentrations of CuSO_4_ (see right). Optical density (*i.e.* OD_600_, y-axis) of each culture is plotted over time (x-axis). Samples are color-coded according to growth relative to that in pH 4.5 for each copper concentration (see asterisks and grey curves). Data in blue and yellow boxes were used for the dose–response curve in B. **(B)** Dose–response curves measuring the effect of increasing media pH (indicated on x-axis) on respiratory growth in the presence (red) or absence (black) of 1333 μM CuSO_4_. Growth relative to that in pH 4.5 is plotted on the y-axis. The mean of three replicates is plotted; error bars represent the standard deviation. **(C and D)** as in **(A and B)**, only examining the combinatorial effects of CuSO_4_ and bafilomycin A1 (a specific inhibitor of vacuolar H^+^−ATPase) on growth in unbuffered YPE media.

These results suggest that the pH gradient across the vacuolar membrane plays a critical role in maintaining optimal copper homeostasis. We therefore examined the effects of increasing media pH on the cellular response to copper (Figure 3A). Because alkaline growth conditions increase vacuolar pH more than cytosolic pH [58,59], they effectively reduce the pH gradient between the two cellular compartments. Consistent with previous findings on dextrose-containing media [60], we find that respiratory growth impairment by alkaline stress, is largely alleviated by supplementing the growth medium with CuSO_4_ (Figure 3A and B). To more directly assess the role of vacuole acidity on copper homeostasis, we applied the small molecule Bafilomycin A1 (BafA), a natural product derived from *Streptomyces griseus* that specifically inhibits vacuolar H^+^−ATPase [61]. BafA strongly inhibited growth of respiring yeast at sub-micromolar concentrations however this inhibition could be suppressed by supplementing the media with CuSO_4_ (Figure 3C and D). These data further establish an important role for the vacuole in controlling cellular copper homeostasis, and moreover identify copper as a major limiting factor under vacuolar stress conditions. Notably, addition of high concentrations of FeSO_4_ also partially suppressed growth inhibition by BafA and masked the effects of CuSO_4_ under these conditions (Additional file 1: Figure S7D). This is consistent with Cu-limitation arising from vacuole de-acidification causing a cellular iron deficiency that negatively impacts growth.

### Human disease genes

Our functional screen was highly sensitive in detecting small Cu-dependent changes in cell fitness. Mutations in non-essential genes that yield subtle phenotypes may be more likely to cause disease, compared to for example, genes required for viability. Therefore, we next examined the genes we identified for sequence homology to human genes associated with disease (see Additional file 2: Table S4 for complete results). Table 1 lists those human disease genes with yeast orthologs, that when deleted, yield respiratory fitness-defects that are rescued by copper supplementation. The diseases associated with these genes include Menkes and Wilson’s disease, both of which are caused by mutations in genes orthologous to yeast *CCC2*, and both of which are well established Cu-related disorders [9,11]. The other diseases in Table 1, typically present with neurologic, musculoskeletal and hematologic features, which show remarkable similarities to clinical phenotypes seen in patients with Menkes' copper deficiency [11]. Many of these disease genes have a role in intracellular trafficking, including several members of the AP-1 adaptor complex, an important component of clathrin-coated vesicles [62]. Interestingly, a combined zebrafish/yeast chemical-genetic screen has also identified this complex as having a conserved role in buffering the effects of copper limitation [63]. Knock-down experiments in human cell lines will determine whether these genes fulfill a similar function in man, and may prompt a closer examination of whether copper plays a role in the disorders listed in Table 1.

**Table 1:** Human disease genes putatively associated with copper imbalance

**Yeast gene**	**Human gene**	**% ident.**	**Subcellular localization**	**Biological function**	**OMIM**	**Clinical phenotype**
*CCC2*	ATP7A	23	Golgi apparatus, Plasma membrane	Cu ion transport across membranes	309400	Menkes disease (copper deficiency)
	ATP7B	24	Golgi apparatus, Mitochondria		277900	Wilson disease (copper overload)
*ADK1*	AK2	54	Mitochondrial inter-membrane space	Energy and nucleotide metabolism	267500	Immunodeficiency, sensorineural deafness
*COX12*	COX6B1	42		Energy metabolism, respiratory chain complex	220110	Encephalopathy, growth retardation, vision loss
*VMA2*	ATP6V1B1	73	Endomembrane, plasma membrane	Vacuolar proton-translocating ATPase	267300	Renal tubular acidosis, sensorineural deafness
*HFA1*	ACACA	38	Mitochondria, cytoplasm	Fatty acid biosynthesis	613933	Encephalopathy, growth retardation, myopathy
*GEF1*	CLCN5	30	Endosome membrane, lysosomal membrane	Chloride channels and ion transporter	300009	Renal tubular disease, kidney stones
	CLCN7	20			166600	Osteosclerosis, multiple fractures, vision loss
*VPS33*	VPS33B	23		Protein transport, membrane fusion	208085	Arthrogryposis, renal dysfunction, cholestasis
*NHX1*	SLC9A9	27	Endosome membrane	pH regulation, ion transport	613410	Autism, seizures
	SLC9A6	26			300243	Mental retardation, seizures, ataxia
*APS1*	AP1S2	51	Golgi apparatus	AP-1 adaptor complex, protein transport, vesicular trafficking	300630	Mental retardation, cerebral calcifications
	AP4S1	26			614067	Spastic paraplegia, mental retardation
*APL2*	AP4B1	22			614066	
*APM1*	AP4M1	24			612936	
*ARL1*	ARL6	39		Protein transport, metal ion binding, membrane trafficking	209900	Mental retardation, obesity, retinopathy
*ARL3*	ARL13B	13			612291	Cerebral malformation, mental retardation
*COG6*	COG6	19		Oligomeric Golgi complex, vesicular transport	606977	Vitamin K deficiency, intracranial bleedings
*APL6*	AP3B1	20		AP-3 adaptor complex, protein transport	608233	Platelet defect, albinism, immunodeficiency
*ERG24*	DHCR7	27	Endoplasmatic reticulum	Cholesterol biosynthesis, sterol metabolism	270400	Mental retardation, congenital malformation
	LBR	27	Nuclear membrane		215140	Skeletal dysplasia, leukocyte disorder

Twenty-one human disease genes which have a yeast orthologue (% identity of human protein in column 3) that, when deleted, produced a respiratory fitness defect that was specifically rescued by copper supplementation. The gene products of the 21 disease genes have distinct functions and subcellular localizations [64], while mutations in these genes are typically associated with neurologic, musculoskeletal and hematologic disease phenotypes resembling those of copper deficiency (http://omim.org/).

### Pharmacological rescue of copper deficiencies

Pharmacological modulation of Cu homeostasis is an attractive therapeutic strategy for a variety of clinical applications. Copper chelating agents are currently the standard treatment to ameliorate copper overloading of affected tissues in Wilson’s disease [11], whereas early treatment of Menkes disease with copper has shown some promise in newborns having mutations that do not completely abrogate ATP7A [65]. Recent work in model systems has also demonstrated that host cell copper metabolism influences RNA virus replication [66], and leveraging copper’s cytotoxic properties is also being explored for cancer therapy, and may be effective in targeted killing of tumor cells [67-72].

**Figure 4:**
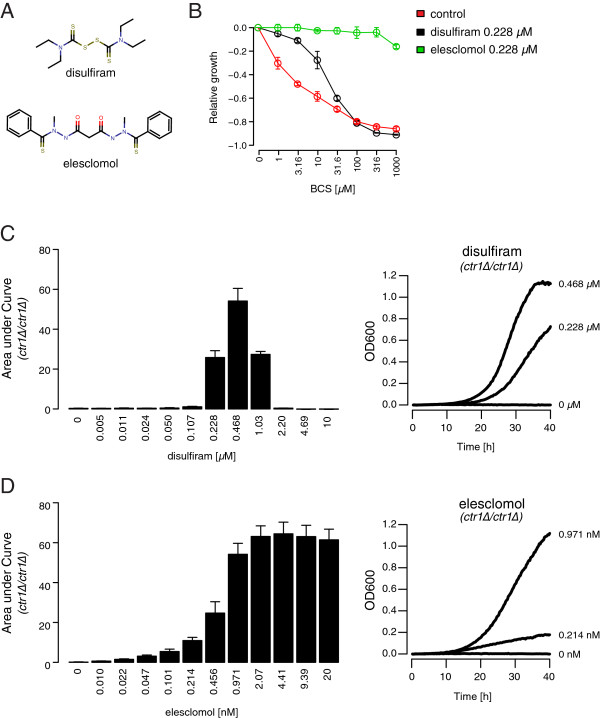
**Pharmacological rescue of Cu-deficiency phenotypes. (A)** Chemical structure of disulfiram (DSF) and elesclomol (ES). **(B)** Dose–response curves measuring the sensitivity of respiring yeast (BY4743 grown in YPE) to increasing concentrations of BCS (indicated on x-axis), in the presence of 0.228 μM DSF (black) or 0.228 μM ES (green). Growth relative to that in the absence of BCS is indicated by the y-axis. The mean of three replicates are plotted; error bars indicate the standard deviation. **(C)** Respiratory growth of the *ctr1Δ/ctr1Δ* strain in response to various concentrations of DSF (indicated on the x-axis). Growth after 40 hours in YPE was measured by area under the curve (y-axis). The mean of 3 replicates is plotted. Error bars indicate the standard deviation. Representative growth curves are shown on the right. **(D)** Similar to **(C)** only involving ES.

The ability of several compounds to increase copper concentrations in different cellular compartments was recently characterized [67]. We examined the effects of two of these compounds (disulfiram and elesclomol; Figure 4A), on respiratory growth in yeast. Consistent with our results in Figure 1, and with their ability to increase intracellular copper concentrations, both DSF and ES conferred significant resistance to the copper chelating agent BCS (Figure 4B). At the equivalent concentration (0.228 μM), ES was more effective than DSF in rescuing BCS-induced growth defects, an observation that is consistent with ES having a stronger effect on intracellular copper concentrations in human cell lines [67].

We next assessed the ability of DSF and ES to rescue the effects of genetically-induced copper deprivation. Our genomic screen identified the *ctr1Δ*/*ctr1Δ* strain, which lacks the primary copper transporter in yeast [42], as being most aided by supplemental copper among the >5000 strains tested (Figure 2D and Additional file 2: Table S1). We found that DSF rescued the severe respiratory defect of this strain in a dose-dependent manner, with the strongest effect observed at 0.468 μM DSF (Figure 4C). ES rescued growth of the *ctr1Δ*/*ctr1Δ* strain as well, and did so over a much broader concentration range, and at sub-nanomolar concentrations (Figure 4D). Comparable to the effects of Cu, both drugs also increased respiratory growth of the parental BY4743 strain (Additional file 1: Figure S8) suggesting that either drug could be used to correct a range of Cu-deficiencies. It is noteworthy that while DSF inhibited growth at higher concentrations, ES-induced growth inhibition was dependent on supplementing the media with Cu (Additional file 1: Figure S8), an observation that is consistent with previous findings [72]. When tested against several strains listed in Table 1, most strains were rescued by both ES and DSF, but interestingly, neither drug rescued the respiratory growth defects of the *gef1Δ*/*gef1Δ* or *ccc2Δ*/*ccc2Δ* strains (Additional file 1: Figure S9), which lack genes specifically required for loading Cu into Fet3. These data suggest that, unlike CuSO_4_ added to the growth media, DSF- and ES-delivered copper require Gef1- and Ccc2-dependent Cu loading into Fet3 to confer enhanced growth.

ES is a promising anti-cancer agent that induces oxidative stress in a copper-dependent manner [67,72]. DSF has been used for the treatment of alcoholism for decades [73] and confers therapeutic value by inhibiting aldehyde dehydrogenase (ALDH), which decreases the alcohol tolerance in patients [74]. DSF has also been shown to perturb vacuolar pH in yeast, and inhibit V-ATPase activity in cell-free assays [75]. Though our results clearly identify copper homeostasis as a rate-limiting effect of these drugs on respiratory growth, further experiments will be required to determine their precise mechanism-of-action. Nevertheless, our data highlight the ability of copper ionophores to potently and precisely tune cellular fitness under copper-limiting conditions. Leveraging the pharmacological tractability of copper homeostasis, though confronted with inherent cytoxicity obstacles, may warrant exploration as a therapeutic strategy for copper- or iron-deficiency in man.

## Conclusions

A systematic screen of the yeast deletion collection was conducted to identify genes that have a role in Cu-dependent respiratory fitness. The screen revealed a complex cellular system involving many genes that were not previously associated with copper homeostasis. The results are consistent with copper limitation producing a secondary iron deficiency that negatively impacts respiratory growth, and with the vacuole playing a vital role in regulating intracellular copper levels. Roughly half of the genes identified in our screen are conserved in man, and many of these are associated with Mendelian diseases, underscoring the potential translation of our findings to human health. Pharmacological agents that increase intracellular copper concentrations can be effective in compensating for perturbations in copper homeostasis.

## Methods

### Reagents

Copper (II) sulfate, tetraethylthiuram disulfide (disulfiram), and bathocuproine disulfonic acid disodium salt (BCS) were purchased from Sigma-Aldrich. Elesclomol was purchased from Selleckchem and bafilomycin A1 was purchased from Enzo Life Sciences. Copper (II) sulfate was dissolved in water and stored at room temperature. BCS, elesclomol, bafilomycin A1, and disulfiram were dissolved in DMSO, aliquoted, and stored at −20°C until use. We used a HP D300 Liquid Dispenser (Tecan) for all experiments involving DMSO-dissolved compounds.

### Media and growth rate analysis

All media was prepared using deionized water. Rich media (YP) consisted of 10 g yeast extract, 10 g bactopeptone per liter. For Figure 3 A and B the YP media was buffered with 25 mM HEPES and pH-balanced as indicated using 1 M NaOH or 12.1 M HCl, to raise or lower the pH, respectively. 25 mM HEPES was also included in the media used for Additional file 1: Figure S1, and we note that addition of the HEPES buffer reduced the respiratory growth rate of yeast. All other experiments were performed using YP media without HEPES. Pre-cultures of the isogenic strains were grown overnight to saturation in rich media supplemented with 2% dextrose. These cultures were then used to inoculate YP media supplemented with carbon sources at 2% as indicated in the Figure legends. Isogenic cultures (100 μl) were inoculated at a concentration of 0.03 OD_600_/ml and grown in 96well microtiter plates at 30°C. The plates were sealed with adhesive plate seals (Thermo Scientific) and holes were poked in the seal for every well using a 21 gauge needle to allow sufficient aeration (cultures using non-fermentable carbon sources only). Optical density was measured every 15 min over the course of several hours (as indicated in graphs) using a GENios microplate reader (Tecan). The growth rate of a strain was calculated as follows: 1) the first 10 OD readings were averaged and subtracted from all OD readings of the corresponding curve in order to set the baseline of the growth curve to zero, 2) the area under the curve (AUC) was then calculated as the sum of all OD readings. In general, 50 and 100 reads (corresponding to 12.5 and 25 hours, respectively) were used for strains grown in fermentative conditions (dextrose and galactose) and in respiratory conditions (ethanol, glycerol, and lactate), respectively. 160 reads (40 hours) was used for Figures 4C and D (*ctr1Δ/ctr1Δ*). A “relative growth” value was calculated as previously described [76], as follows: (AUC_condition_ – AUC_control_)/AUC_control_; where AUC_control_ represents the growth rate of the reference condition (marked with an asterisk in all Figures) that was assayed on the same microtiter plate.

### Deletion pool construction and growth conditions

The homozygous deletion pool was constructed as described [30] and stored in aliquots at −80°C. For all screening experiments, aliquots of the pool were thawed and diluted in YP media plus a carbon source (2% of dextrose, ethanol, glycerol or lactate) to a concentration of 0.04 OD_600_/ml. 700 μl of this cell suspension was then aliquoted into every well of a 48well plate and copper sulfate was then added (from an 500 mM stock solution in water) to a final concentration of 500 μM. The plates were sealed with adhesive plate seals and holes were poked for every well to allow sufficient aeration. The plates were incubated at 30°C with vigorous shaking and optical density was measured every 15 min over the course of the experiment. Each sample was grown for precisely 9 generations. Cells were maintained in logarithmic phase by robotically diluting cultures every three doublings using a Packard Multiprobe II four-probe liquid-handling system (PerkinElmer, Wellesley, California, United States). Experiments were performed in triplicate. After reaching 9 pool generations 600 μl of the cultures were harvested, pelleted by centrifugation and snap frozen in liquid nitrogen. The pellets were kept at −80°C until further processing (genomic DNA preparation, PCR, and microarray hybridization, see below).

To construct the pool of respiration deficient mutants we first analyzed samples from the YPD, YPE, YPG, and YPL experiments where no additional copper was added to the media. Strains with a median of log2-transformed raw fluorescence units < 7 in the combined YPE, YPG, and YPL samples and a median of log2-transformed raw fluorescence units > 8 in the YPD samples were selected. This identified 313 strains, to which we added an additional 18 strains of interest (Additional file 2: Table S1). The 331 strains were streaked from frozen stocks onto YPD agar plates, grown for 3 days at 30°C, and then pooled and stored in aliquots at −80°C. This pool was assayed as described above for the complete homozygous deletion pool.

### Genomic DNA preparation, PCR, and microarray hybridization

Genomic DNA preparation, PCR amplification of molecular tags, and microarray hybridization were as described [77]. Genomic DNA was extracted from cell pellets using the YeaStar Genomic DNA kit from Zymo Research (D2002). The relative abundance of each strain was then assessed by amplifying and hybridizing their molecular barcodes to a Genflex Tag 16 k array (Affymetrix).

### Identification of strains exhibiting increased or decreased Cu-dependent growth

To quantify Cu-dependent growth of each deletion strain, we calculated a “Copper Response Score” (CRS) for each strain in each of the four respiratory pool assays; *i.e.* the homozygous diploid (HD) deletion pool in ethanol-, glycerol-, or lactate-containing media (YPE, YPG, and YPL), and the respiratory-deficient (RD) deletion pool in ethanol-containing medium (YPE*). The majority of strains carry two tags that hybridize to the array, an “uptag” and a “downtag” [30]. From each array, we extracted the fluorescence intensity values for every uptag and downtag associated with the 5050 strains in the HD pool, or in the case of the RD pool, the up- and downtags associated with the 331 strains in that pool (Additional file 2: Table S1). In total, this amounted to 9937, and 675 tags, respectively. The number of tags is not exactly twice the number of strains because some strains contain only one tag. These raw fluorescence values were then log2-transformed, and quantile-normalized using the normalize.quantiles function of the preprocessCore package in R. Quantile normalization was performed on four separate groups of tags; uptags from the HD pool samples, downtags from the HD pool samples, uptags from the RD pool samples, and downtags from the RD pool samples. Eight experimental conditions (*i.e.* the four respiratory pool assays +/− CuSO_4_) were performed in triplicate, and from this set of triplicate measurements, we calculated the mean of the normalized fluorescence value for every tag. For each of the four respiratory pool assays, the mean values from CuSO_4_-untreated samples were then subtracted from the mean values of the CuSO_4_-treated samples. This value is the CRS. In cases where a strain contained two tags (*i.e.* the vast majority of strains), the CRS was derived from that tag having the greatest difference between Cu-untreated and -treated samples (*i.e.* the maximum absolute value of the Δmeans for the uptag and downtag). A moderated t-statistic was used to define differential effects of Cu-treatment in the four respiratory pools using the R package LIMMA [78,79], and the derived p-values were further converted to q-values using Benjamini & Hochberg False Discovery Rate (FDR) correction [80]. 105 strains that exhibited a significant (q-value < 0.05) and large (CRS > 1.5) response to copper in at least one of the four respiratory pool assays (YPG, YPL, YPE, YPE*) were defined as having an increased Cu-boost, and 79 strains that had a significant (q-value < 0.05) and small (CRS < −1.5) response to copper in at least one of these assays were defined as having a decreased Cu-boost. All instances where these criteria are met are illustrated by the filled circles in Additional file 1: Figure S3 and S4. The complete list of CRSs is provided in Additional file 2: Table S1. CEL files are available via the NCBI's Gene Expression Omnibus [35] under accession number GSE47175.

### Hierarchical clustering

For the cluster analysis in Figure 2B, we calculated the Cu-induced fold change of the 184 (79 + 105) strains identified above, in each of the 12 individual replicate experiments involving the HD pool (YPE, YPG, YPL, and YPD), and applied hierarchical clustering to these data. Cu-induced fold change was calculated as (log2 (YPx + Cu)/(YPx-Cu)_mean_), where x is one of the 4 carbon sources (ethanol, glycerol, lactate, dextrose) tested. Fold changes of uptag and downtag were averaged. Clustering along both experiment and gene axes was performed on these values using the TIBCO Spotfire software platform.

### GO term enrichment analysis

To identify enriched Gene Ontology terms among the genes that were identified in this study as enhancing or diminishing the response to copper we used the Biological Networks Gene Ontology plugin (BiNGO, version 2.4.4) for Cytoscape (version 2.8.3). Both gene lists (consisting of 93 and 73 open-reading frames, respectively), were analyzed separately using a Hypergeometric test with Benjamini & Hochberg False Discovery Rate (FDR) correction and a significance level of < 0.05. As a reference set we used the list of 4913 unique open-reading frames represented in the complete pool of 5050 homozygous deletion strains. We applied a force-directed layout to minimize the number of crossing edges and to enhance readability of node labels. Nodes were colored based on the degree of significance of overrepresentation. Uncolored nodes are not overrepresented, but they are the parents of overrepresented categories further down. The yellow and orange nodes represent terms with significant enrichment, with darker orange representing a higher degree of significance, as shown by the color legend panel in Figures 2C and Additional file 1: Figure S6. The size of each node is proportional to the number of genes with that term in the query set.

## Availability of supporting data

The array data supporting the results of this article have been deposited in NCBI's Gene Expression Omnibus [35] and are accessible through GEO Series accession number GSE47175 (http://www.ncbi.nlm.nih.gov/geo/query/acc.cgi?acc=GSE47175).

## Abbreviations

DSF: Disulfiram; ES: Elesclomol; BCS: Bathocuproine disulphonic acid; Cu: Copper; YPD: Yeast extract, peptone, dextrose; YPE: Yeast extract, peptone, ethanol; YPG: Yeast extract, peptone, glycerol; YPL: Yeast extract, peptone, lactate; AUC: Area under curve; BafA: Bafilomycin A; CRS: Copper response score.

## Competing interests

The authors declare that they have no competing interests.

## Authors’ contributions

US, CS, and RPS conceived and designed the experiments, analyzed the data, and wrote the paper; WX analyzed the data; RWD provided insight and advice; MJP developed the robotics for deletion pool screening; US, SS, AMA, AC, and RPS performed experiments. All authors read and approved the final manuscript.

## Supplementary Material

Additional file 1: Figures S1 Effect of copper in media containing different carbon sources, **Figures S2.** Deletion pool results for the *fsh1Δ*/*fsh1Δ* deletion strain in different carbon sources, **Figures S3.** Deletion pool results for 105 strains in which the beneficial effects of CuSO_4_ were greater compared to other strains in the pool, **Figures S4.** Deletion pool results for 79 strains in which the beneficial effects of CuSO_4_ were smaller than other strains in the pool. **Figures S5.** Relationship between Copper Response Score and respiratory fitness, **Figures S6.** Network representation of Gene Ontology (GO) categories that are significantly over-represented among strains exhibiting diminished or enhanced Cu-dependent growth, **Figures S7.** Effects of FeSO_4_ on Cu-dependent growth, **Figures S8.** Growth kinetics of elesclomol and disulfiram-treated cultures in the presence and absence of high concentrations of CuSO_4_, **Figures S9.** Respiratory growth of eleven deletion strains listed in Table 1, in CuSO_4_, disulfiram, or elesclomol. Click here for file

Additional file 2: Tables S1 A summary of the strains and their Copper Response Scores, **Tables S2.** Significantly enriched Gene Ontology categories among the 79 strains in which Cu’s positive effect on respiratory growth was quantitatively reduced, **Tables S3.** Significantly enriched Gene Ontology categories among the 105 strains in which Cu’s positive effects were substantively greater than other strains in the pool, **Tables S4.** 151 human orthologs of 81 yeast genes identified in this study. Click here for file

## References

[B1] Kim BE Nevitt T Thiele DJ Mechanisms for copper acquisition, distribution and regulation Nat Chem Biol 2008 15 3 176 185 10.1038/nchembio.72 18277979

[B2] Horn D Barrientos A Mitochondrial copper metabolism and delivery to cytochrome c oxidase IUBMB Life 2008 15 7 421 429 10.1002/iub.50 18459161 PMC2864105

[B3] Valentine JS Doucette PA Zittin Potter S Copper-zinc superoxide dismutase and amyotrophic lateral sclerosis Annu Rev Biochem 2005 15 563 593 10.1146/annurev.biochem.72.121801.161647 15952898

[B4] Furukawa Y Torres AS O'Halloran TV Oxygen-induced maturation of SOD1: a key role for disulfide formation by the copper chaperone CCS Embo J 2004 15 14 2872 2881 10.1038/sj.emboj.7600276 15215895 PMC1150991

[B5] Hellman NE Gitlin JD Ceruloplasmin metabolism and function Annu Rev Nutr 2002 15 439 458 10.1146/annurev.nutr.22.012502.114457 12055353

[B6] Stearman R Yuan DS Yamaguchi-Iwai Y Klausner RD Dancis A A permease-oxidase complex involved in high-affinity iron uptake in yeast Science 1996 15 5255 1552 1557 10.1126/science.271.5255.1552 8599111

[B7] De Silva DM Askwith CC Eide D Kaplan J The FET3 gene product required for high affinity iron transport in yeast is a cell surface ferroxidase J Biol Chem 1995 15 3 1098 1101 10.1074/jbc.270.3.1098 7836366

[B8] Robinson NJ Winge DR Copper metallochaperones Annu Rev Biochem 2010 15 537 562 10.1146/annurev-biochem-030409-143539 20205585 PMC3986808

[B9] Ala A Walker AP Ashkan K Dooley JS Schilsky ML Wilson's disease Lancet 2007 15 9559 397 408 10.1016/S0140-6736(07)60196-2 17276780

[B10] Menkes JH Alter M Steigleder GK Weakley DR Sung JH A sex-linked recessive disorder with retardation of growth, peculiar hair, and focal cerebral and cerebellar degeneration Pediatrics 1962 15 764 779 14472668

[B11] De Bie P Muller P Wijmenga C Klomp LW Molecular pathogenesis of Wilson and Menkes disease: correlation of mutations with molecular defects and disease phenotypes J Med Genet 2007 15 11 673 688 10.1136/jmg.2007.052746 17717039 PMC2752173

[B12] Horng YC Leary SC Cobine PA Young FB George GN Shoubridge EA Winge DR Human Sco1 and Sco2 function as copper-binding proteins J Biol Chem 2005 15 40 34113 34122 10.1074/jbc.M506801200 16091356

[B13] Leary SC Cobine PA Kaufman BA Guercin GH Mattman A Palaty J Lockitch G Winge DR Rustin P Horvath R Shoubridge EA The human cytochrome c oxidase assembly factors SCO1 and SCO2 have regulatory roles in the maintenance of cellular copper homeostasis Cell Metab 2007 15 1 9 20 10.1016/j.cmet.2006.12.001 17189203

[B14] Leary SC Kaufman BA Pellecchia G Guercin GH Mattman A Jaksch M Shoubridge EA Human SCO1 and SCO2 have independent, cooperative functions in copper delivery to cytochrome c oxidase Hum Mol Genet 2004 15 17 1839 1848 10.1093/hmg/ddh197 15229189

[B15] Bestwick M Jeong MY Khalimonchuk O Kim H Winge DR Analysis of Leigh syndrome mutations in the yeast SURF1 homolog reveals a new member of the cytochrome oxidase assembly factor family Mol Cell Biol 2010 15 18 4480 4491 10.1128/MCB.00228-10 20624914 PMC2937524

[B16] Valnot I Osmond S Gigarel N Mehaye B Amiel J Cormier-Daire V Munnich A Bonnefont JP Rustin P Rotig A Mutations of the SCO1 gene in mitochondrial cytochrome c oxidase deficiency with neonatal-onset hepatic failure and encephalopathy Am J Hum Genet 2000 15 5 1104 1109 11013136 10.1016/s0002-9297(07)62940-1 PMC1288552

[B17] Horvath R Lochmuller H Stucka R Yao J Shoubridge EA Kim SH Gerbitz KD Jaksch M Characterization of human SCO1 and COX17 genes in mitochondrial cytochrome-c-oxidase deficiency Biochem Biophys Res Commun 2000 15 2 530 533 10.1006/bbrc.2000.3495 11027508

[B18] Papadopoulou LC Sue CM Davidson MM Tanji K Nishino I Sadlock JE Krishna S Walker W Selby J Glerum DM Coster RV Lyon G Scalais E Lebel R Kaplan P Shanske S De Vivo DC Bonilla E Hirano M DiMauro S Schon EA Fatal infantile cardioencephalomyopathy with COX deficiency and mutations in SCO2, a COX assembly gene Nat Genet 1999 15 3 333 337 10.1038/15513 10545952

[B19] Jaksch M Ogilvie I Yao J Kortenhaus G Bresser HG Gerbitz KD Shoubridge EA Mutations in SCO2 are associated with a distinct form of hypertrophic cardiomyopathy and cytochrome c oxidase deficiency Hum Mol Genet 2000 15 5 795 801 10.1093/hmg/9.5.795 10749987

[B20] Desai V Kaler SG Role of copper in human neurological disorders Am J Clin Nutr 2008 15 3 855S 858S 18779308 10.1093/ajcn/88.3.855S

[B21] Strozyk D Launer LJ Adlard PA Cherny RA Tsatsanis A Volitakis I Blennow K Petrovitch H White LR Bush AI Zinc and copper modulate Alzheimer Abeta levels in human cerebrospinal fluid Neurobiol Aging 2009 15 7 1069 1077 10.1016/j.neurobiolaging.2007.10.012 18068270 PMC2709821

[B22] Fox JH Kama JA Lieberman G Chopra R Dorsey K Chopra V Volitakis I Cherny RA Bush AI Hersch S Mechanisms of copper ion mediated Huntington's disease progression PLoS One 2007 15 3 e334 10.1371/journal.pone.0000334 17396163 PMC1828629

[B23] Rasia RM Bertoncini CW Marsh D Hoyer W Cherny D Zweckstetter M Griesinger C Jovin TM Fernandez CO Structural characterization of copper(II) binding to alpha-synuclein: Insights into the bioinorganic chemistry of Parkinson's disease Proc Natl Acad Sci U S A 2005 15 12 4294 4299 10.1073/pnas.0407881102 15767574 PMC555498

[B24] De Freitas J Wintz H Kim JH Poynton H Fox T Vulpe C Yeast, a model organism for iron and copper metabolism studies Biometals 2003 15 1 185 197 10.1023/A:1020771000746 12572678

[B25] Askwith C Kaplan J Iron and copper transport in yeast and its relevance to human disease Trends Biochem Sci 1998 15 4 135 138 10.1016/S0968-0004(98)01192-X 9584616

[B26] Diaz-Ruiz R Uribe-Carvajal S Devin A Rigoulet M Tumor cell energy metabolism and its common features with yeast metabolism Biochim Biophys Acta 2009 15 2 252 265 19682552 10.1016/j.bbcan.2009.07.003

[B27] Rossignol R Gilkerson R Aggeler R Yamagata K Remington SJ Capaldi RA Energy substrate modulates mitochondrial structure and oxidative capacity in cancer cells Cancer Res 2004 15 3 985 993 10.1158/0008-5472.CAN-03-1101 14871829

[B28] St Onge R Schlecht U Scharfe C Evangelista M Forward chemical genetics in yeast for discovery of chemical probes targeting metabolism Molecules 2012 15 11 13098 13115 23128089 10.3390/molecules171113098 PMC3539408

[B29] Hillenmeyer ME Fung E Wildenhain J Pierce SE Hoon S Lee W Proctor M St Onge RP Tyers M Koller D Altman RB Davis RW Nislow C Giaever G The chemical genomic portrait of yeast: uncovering a phenotype for all genes Science 2008 15 5874 362 365 10.1126/science.1150021 18420932 PMC2794835

[B30] Lee W St Onge RP Proctor M Flaherty P Jordan MI Arkin AP Davis RW Nislow C Giaever G Genome-wide requirements for resistance to functionally distinct DNA-damaging agents PLoS Genet 2005 15 2 e24 10.1371/journal.pgen.0010024 16121259 PMC1189734

[B31] Steinmetz LM Scharfe C Deutschbauer AM Mokranjac D Herman ZS Jones T Chu AM Giaever G Prokisch H Oefner PJ Davis RW Systematic screen for human disease genes in yeast Nat Genet 2002 15 4 400 404 12134146 10.1038/ng929

[B32] Giaever G Chu AM Ni L Connelly C Riles L Veronneau S Dow S Lucau-Danila A Anderson K Andre B Arkin AP Astromoff A El-Bakkoury M Bangham R Benito R Brachat S Campanaro S Curtiss M Davis K Deutschbauer A Entian KD Flaherty P Foury F Garfinkel DJ Gerstein M Gotte D Güldener U Hegemann JH Hempel S Herman Z Functional profiling of the Saccharomyces cerevisiae genome Nature 2002 15 6896 387 391 10.1038/nature00935 12140549

[B33] Winzeler EA Shoemaker DD Astromoff A Liang H Anderson K Andre B Bangham R Benito R Boeke JD Bussey H Chu AM Connelly C Davis K Dietrich F Dow SW El Bakkoury M Foury F Friend SH Gentalen E Giaever G Hegemann JH Jones T Laub M Liao H Liebundguth N Lockhart DJ Lucau-Danila A Lussier M M'Rabet N Menard P Functional characterization of the S. cerevisiae genome by gene deletion and parallel analysis Science 1999 15 5429 901 906 10.1126/science.285.5429.901 10436161

[B34] Jo WJ Loguinov A Chang M Wintz H Nislow C Arkin AP Giaever G Vulpe CD Identification of genes involved in the toxic response of Saccharomyces cerevisiae against iron and copper overload by parallel analysis of deletion mutants Toxicol Sci 2008 15 1 140 151 17785683 10.1093/toxsci/kfm226

[B35] Edgar R Domrachev M Lash AE Gene Expression Omnibus: NCBI gene expression and hybridization array data repository Nucleic Acids Res 2002 15 1 207 210 10.1093/nar/30.1.207 11752295 PMC99122

[B36] Huang J Hu N Goldstein AM Emmert-Buck MR Tang ZZ Roth MJ Wang QH Dawsey SM Han XY Ding T Li G Giffen C Taylor PR High frequency allelic loss on chromosome 17p13.3-p11.1 in esophageal squamous cell carcinomas from a high incidence area in northern China Carcinogenesis 2000 15 11 2019 2026 10.1093/carcin/21.11.2019 11062163

[B37] Schultz DC Vanderveer L Berman DB Hamilton TC Wong AJ Godwin AK Identification of two candidate tumor suppressor genes on chromosome 17p13.3 Cancer Res 1996 15 9 1997 2002 8616839

[B38] Maere S Heymans K Kuiper M BiNGO: a Cytoscape plugin to assess overrepresentation of gene ontology categories in biological networks Bioinformatics 2005 15 16 3448 3449 10.1093/bioinformatics/bti551 15972284

[B39] Askwith C Eide D Van Ho A Bernard PS Li L Davis-Kaplan S Sipe DM Kaplan J The FET3 gene of S. cerevisiae encodes a multicopper oxidase required for ferrous iron uptake Cell 1994 15 2 403 410 10.1016/0092-8674(94)90346-8 8293473

[B40] Arredondo M Nunez MT Iron and copper metabolism Mol Aspects Med 2005 15 4–5 313 327 16112186 10.1016/j.mam.2005.07.010

[B41] Dancis A Yuan DS Haile D Askwith C Eide D Moehle C Kaplan J Klausner RD Molecular characterization of a copper transport protein in S. cerevisiae: an unexpected role for copper in iron transport Cell 1994 15 2 393 402 10.1016/0092-8674(94)90345-X 8293472

[B42] Dancis A Haile D Yuan DS Klausner RD The Saccharomyces cerevisiae copper transport protein (Ctr1p). Biochemical characterization, regulation by copper, and physiologic role in copper uptake J Biol Chem 1994 15 41 25660 25667 7929270

[B43] Gaxiola RA Yuan DS Klausner RD Fink GR The yeast CLC chloride channel functions in cation homeostasis Proc Natl Acad Sci U S A 1998 15 7 4046 4050 10.1073/pnas.95.7.4046 9520490 PMC19960

[B44] Reinders J Zahedi RP Pfanner N Meisinger C Sickmann A Toward the complete yeast mitochondrial proteome: multidimensional separation techniques for mitochondrial proteomics J Proteome Res 2006 15 7 1543 1554 10.1021/pr050477f 16823961

[B45] Sickmann A Reinders J Wagner Y Joppich C Zahedi R Meyer HE Schonfisch B Perschil I Chacinska A Guiard B Rehling P Pfanner N Meisinger C The proteome of Saccharomyces cerevisiae mitochondria Proc Natl Acad Sci U S A 2003 15 23 13207 13212 10.1073/pnas.2135385100 14576278 PMC263752

[B46] Cobine PA Ojeda LD Rigby KM Winge DR Yeast contain a non-proteinaceous pool of copper in the mitochondrial matrix J Biol Chem 2004 15 14 14447 14455 10.1074/jbc.M312693200 14729672

[B47] Horng YC Cobine PA Maxfield AB Carr HS Winge DR Specific copper transfer from the Cox17 metallochaperone to both Sco1 and Cox11 in the assembly of yeast cytochrome C oxidase J Biol Chem 2004 15 34 35334 35340 10.1074/jbc.M404747200 15199057

[B48] Glerum DM Shtanko A Tzagoloff A SCO1 and SCO2 act as high copy suppressors of a mitochondrial copper recruitment defect in Saccharomyces cerevisiae J Biol Chem 1996 15 34 20531 20535 10.1074/jbc.271.34.20531 8702795

[B49] Glerum DM Shtanko A Tzagoloff A Characterization of COX17, a yeast gene involved in copper metabolism and assembly of cytochrome oxidase J Biol Chem 1996 15 24 14504 14509 10.1074/jbc.271.24.14504 8662933

[B50] Klionsky DJ Herman PK Emr SD The fungal vacuole: composition, function, and biogenesis Microbiol Rev 1990 15 3 266 292 2215422 10.1128/mr.54.3.266-292.1990 PMC372777

[B51] Li SC Kane PM The yeast lysosome-like vacuole: endpoint and crossroads Biochim Biophys Acta 2009 15 4 650 663 10.1016/j.bbamcr.2008.08.003 18786576 PMC2906225

[B52] Portnoy ME Schmidt PJ Rogers RS Culotta VC Metal transporters that contribute copper to metallochaperones in Saccharomyces cerevisiae Mol Genet Genomics 2001 15 5 873 882 10.1007/s004380100482 11523804

[B53] Rees EM Lee J Thiele DJ Mobilization of intracellular copper stores by the ctr2 vacuolar copper transporter J Biol Chem 2004 15 52 54221 54229 10.1074/jbc.M411669200 15494390

[B54] Yuan DS Dancis A Klausner RD Restriction of copper export in Saccharomyces cerevisiae to a late Golgi or post-Golgi compartment in the secretory pathway J Biol Chem 1997 15 41 25787 25793 10.1074/jbc.272.41.25787 9325307

[B55] Bellemare DR Shaner L Morano KA Beaudoin J Langlois R Labbe S Ctr6, a vacuolar membrane copper transporter in Schizosaccharomyces pombe J Biol Chem 2002 15 48 46676 46686 10.1074/jbc.M206444200 12244050

[B56] Bertinato J Swist E Plouffe LJ Brooks SP L'Abbe MR Ctr2 is partially localized to the plasma membrane and stimulates copper uptake in COS-7 cells Biochem J 2008 15 3 731 740 10.1042/BJ20071025 17944601

[B57] Qi J Han A Yang Z Li C Metal-sensing transcription factors Mac1p and Aft1p coordinately regulate vacuolar copper transporter CTR2 in Saccharomyces cerevisiae Biochem Biophys Res Commun 2012 15 2 424 428 10.1016/j.bbrc.2012.05.150 22683637

[B58] Orij R Urbanus ML Vizeacoumar FJ Giaever G Boone C Nislow C Brul S Smits GJ Genome-wide analysis of intracellular pH reveals quantitative control of cell division rate by pH(c) in Saccharomyces cerevisiae Genome Biol 2012 15 9 R80 10.1186/gb-2012-13-9-r80 23021432 PMC3506951

[B59] Brett CL Kallay L Hua Z Green R Chyou A Zhang Y Graham TR Donowitz M Rao R Genome-wide analysis reveals the vacuolar pH-stat of Saccharomyces cerevisiae PLoS One 2011 15 3 e17619 10.1371/journal.pone.0017619 21423800 PMC3056714

[B60] Serrano R Bernal D Simon E Arino J Copper and iron are the limiting factors for growth of the yeast Saccharomyces cerevisiae in an alkaline environment J Biol Chem 2004 15 19 19698 19704 10.1074/jbc.M313746200 14993228

[B61] Huss M Wieczorek H Inhibitors of V-ATPases: old and new players J Exp Biol 2009 15 Pt 3 341 346 19151208 10.1242/jeb.024067

[B62] Kirchhausen T Three ways to make a vesicle Nat Rev Mol Cell Biol 2000 15 3 187 198 10.1038/35043117 11252894

[B63] Ishizaki H Spitzer M Wildenhain J Anastasaki C Zeng Z Dolma S Shaw M Madsen E Gitlin J Marais R Tyers M Patton EE Combined zebrafish-yeast chemical-genetic screens reveal gene-copper-nutrition interactions that modulate melanocyte pigmentation Dis Model Mech 2010 15 9–10 639 651 20713646 10.1242/dmm.005769 PMC2938393

[B64] Maglott D Ostell J Pruitt KD Tatusova T Entrez Gene: gene-centered information at NCBI Nucleic Acids Res 2011 15 Database issue D52 D57 21115458 10.1093/nar/gkq1237 PMC3013746

[B65] Kaler SG Holmes CS Goldstein DS Tang J Godwin SC Donsante A Liew CJ Sato S Patronas N Neonatal diagnosis and treatment of Menkes disease N Engl J Med 2008 15 6 605 614 10.1056/NEJMoa070613 18256395 PMC3477514

[B66] Sasvari Z Kovalev N Nagy PD The GEF1 proton-chloride exchanger affects tombusvirus replication via regulation of copper metabolism in yeast J Virol 2013 15 3 1800 1810 10.1128/JVI.02003-12 23192874 PMC3554144

[B67] Nagai M Vo NH Shin Ogawa L Chimmanamada D Inoue T Chu J Beaudette-Zlatanova BC Lu R Blackman RK Barsoum J Koya K Wada Y The oncology drug elesclomol selectively transports copper to the mitochondria to induce oxidative stress in cancer cells Free Radic Biol Med 2012 15 10 2142 2150 10.1016/j.freeradbiomed.2012.03.017 22542443

[B68] Cen D Brayton D Shahandeh B Meyskens FL Jr Farmer PJ Disulfiram facilitates intracellular Cu uptake and induces apoptosis in human melanoma cells J Med Chem 2004 15 27 6914 6920 10.1021/jm049568z 15615540

[B69] Cen D Gonzalez RI Buckmeier JA Kahlon RS Tohidian NB Meyskens FL Jr Disulfiram induces apoptosis in human melanoma cells: a redox-related process Mol Cancer Ther 2002 15 3 197 204 12467214

[B70] Chen D Cui QC Yang H Dou QP Disulfiram, a clinically used anti-alcoholism drug and copper-binding agent, induces apoptotic cell death in breast cancer cultures and xenografts via inhibition of the proteasome activity Cancer Res 2006 15 21 10425 10433 10.1158/0008-5472.CAN-06-2126 17079463

[B71] Liu P Brown S Goktug T Channathodiyil P Kannappan V Hugnot JP Guichet PO Bian X Armesilla AL Darling JL Wang W Cytotoxic effect of disulfiram/copper on human glioblastoma cell lines and ALDH-positive cancer-stem-like cells Br J Cancer 2012 15 9 1488 1497 10.1038/bjc.2012.442 23033007 PMC3493777

[B72] Blackman RK Cheung-Ong K Gebbia M Proia DA He S Kepros J Jonneaux A Marchetti P Kluza J Rao PE Wada Y Giaever G Nislow C Mitochondrial electron transport is the cellular target of the oncology drug elesclomol PLoS One 2012 15 1 e29798 10.1371/journal.pone.0029798 22253786 PMC3256171

[B73] Suh JJ Pettinati HM Kampman KM O'Brien CP The status of disulfiram: a half of a century later J Clin Psychopharmacol 2006 15 3 290 302 10.1097/01.jcp.0000222512.25649.08 16702894

[B74] Deitrich RA Erwin VG Mechanism of the inhibition of aldehyde dehydrogenase in vivo by disulfiram and diethyldithiocarbamate Mol Pharmacol 1971 15 3 301 307 4328422

[B75] Johnson RM Allen C Melman SD Waller A Young SM Sklar LA Parra KJ Identification of inhibitors of vacuolar proton-translocating ATPase pumps in yeast by high-throughput screening flow cytometry Anal Biochem 2010 15 2 203 211 10.1016/j.ab.2009.12.020 20018164 PMC2853757

[B76] Schlecht U Miranda M Suresh S Davis RW St Onge RP Multiplex assay for condition-dependent changes in protein-protein interactions Proc Natl Acad Sci U S A 2012 15 23 9213 9218 10.1073/pnas.1204952109 22615397 PMC3384208

[B77] Hoon S Smith AM Wallace IM Suresh S Miranda M Fung E Proctor M Shokat KM Zhang C Davis RW Giaever G St Onge RP Nislow C An integrated platform of genomic assays reveals small-molecule bioactivities Nat Chem Biol 2008 15 8 498 506 10.1038/nchembio.100 18622389

[B78] Smyth GK Yang YH Speed T Statistical issues in cDNA microarray data analysis Methods Mol Biol 2003 15 111 136 12710670 10.1385/1-59259-364-X:111

[B79] Smyth GK Gentleman R, Carey V, Huber W, Irizarry R, Dudoit S Limma: linear models for microarray data Bioinformatics and Computational Biology Solutions Using R and Bioconductor 2005 New York, NY.: Springer 397 420

[B80] Benjamini Y Hochberg Y Controlling the false discovery rate: a practical and powerful approach to multiple testing Journal of the Royal Statistical Society SeriesB 1995 15 289 300

