# Supplementary material for: A functional screen for copper homeostasis genes identifies a pharmacologically tractable cellular system
Source: BMC Genomics. 2014 Apr 5;15:263. doi: 10.1186/1471-2164-15-263 (PMC4023593; doi:10.1186/1471-2164-15-263)
Supplement: Additional file 1: Figures S1 — Effect of copper in media containing different carbon sources, Figures S2. Deletion pool results for the fsh1Δ/fsh1Δ deletion strain in different carbon sources, Figures S3. Deletion pool results for 105 strains in which the beneficial effects of CuSO4 were greater compared to other strains in the pool, Figures S4. Deletion pool results for 79 strains in which the beneficial effects of CuSO4 were smaller than other strains in the pool. Figures S5. Relationship between Copper Response Score and respiratory fitness, Figures S6. Network representation of Gene Ontology (GO) categories that are significantly over-represented among strains exhibiting diminished or enhanced Cu-dependent growth, Figures S7. Effects of FeSO4 on Cu-dependent growth, Figures S8. Growth kinetics of elesclomol and disulfiram-treated cultures in the presence and absence of high concentrations of CuSO4, Figures S9. Respiratory growth of eleven deletion strains listed in Table 1, in CuSO4, disulfiram, or elesclomol. [file 1471-2164-15-263-S1.pdf]

Figure S1

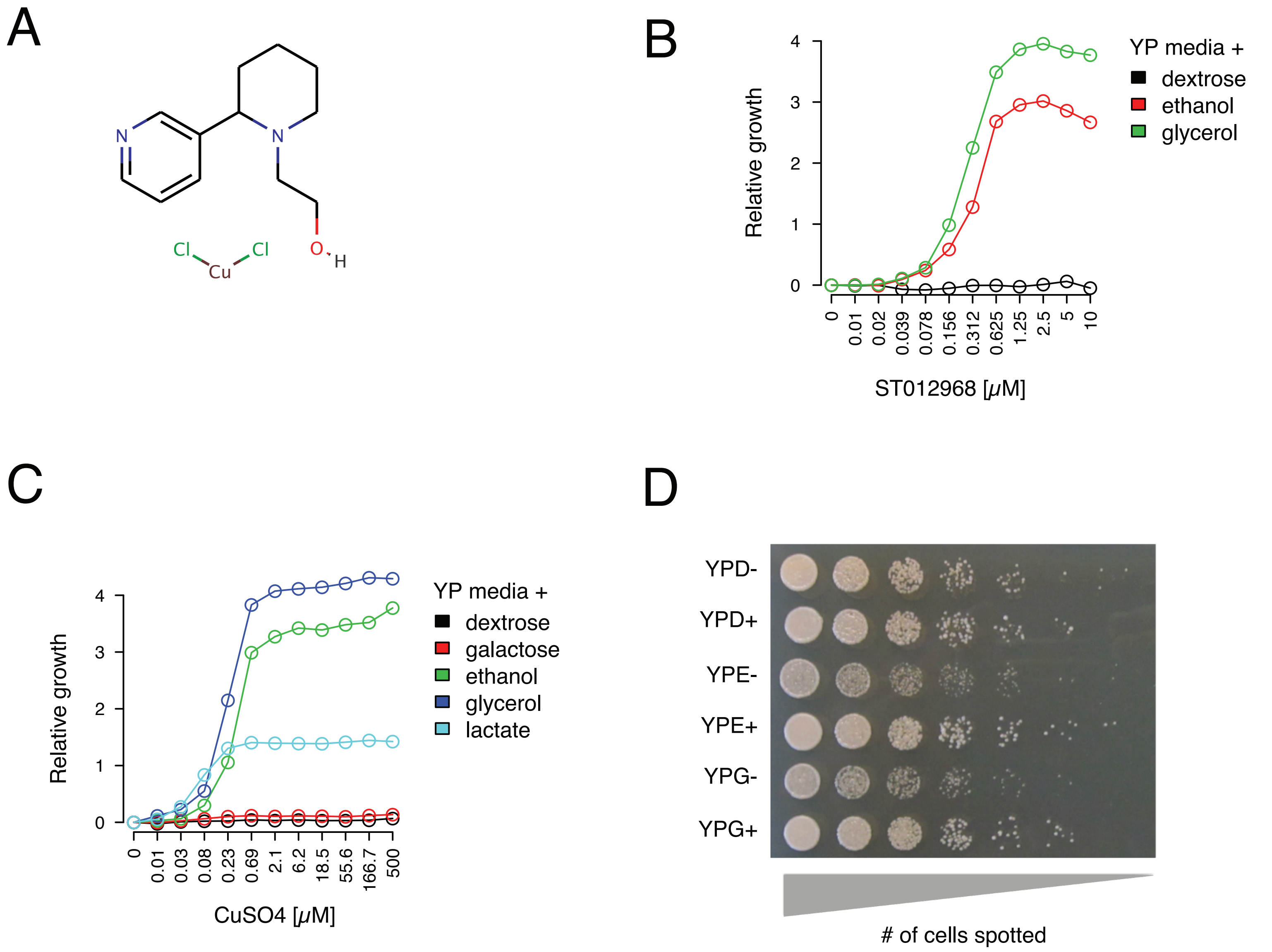

A
N
N
Cl
Cl
Cu
O
H
B
Relative growth
4
3
2
1
0
0
0.01
0.02
0.039
0.078
0.156
0.312
0.625
1.25
2.5
5
10
ST012968 [μM]
YP media +
dextrose
ethanol
glycerol
C
Relative growth
4
3
2
1
0
0
0.01
0.03
0.08
0.23
0.69
2.1
6.2
18.5
55.6
166.7
500
CuSO4 [μM]
YP media +
dextrose
galactose
ethanol
glycerol
lactate
D
YPD-
YPD+
YPE-
YPE+
YPG-
YPG+
# of cells spotted

Figure S1. Copper increases (i.e. 'boosts') growth on/in media containing non-fermentable carbon sources. (A) Chemical structure of ST012968 (Pubchem CID 24207270), which was identified in a phenotypic screen for compounds that increase respiratory growth in yeast. (B) Dose-dependent effect of ST012968 on growth in various carbon sources. Wild-type yeast (BY4743) was grown in YP media supplemented with dextrose (black), ethanol (red), or glycerol (green), in the presence of multiple concentrations of ST012968 (indicated on the x-axis). Growth relative to that in the absence of the compound is indicated by the y-axis. (C) Dose-dependent effect of copper sulfate (CuSO4) on growth in various carbon sources. Wild-type yeast (BY4743) was grown in YP media containing the fermentable carbon sources dextrose (black) or galactose (red), or the non-fermentable carbon sources ethanol (green), glycerol (dark blue), or lactate (light blue), in the presence of multiple concentrations of CuSO4 (indicated on the x-axis). Growth relative to that in the absence of CuSO4 is indicated by the y-axis. In both (B) and (C), the Cu-dependent increase in relative growth was larger than that observed in Figure 1 due to inclusion of HEPES buffer in the growth medium, which was found to reduce the baseline respiratory growth rate of yeast (see Methods). (D) 500 µM CuSO4 does not have a large impact on yeast viability. Wild-type yeast was grown in various media (indicated on the left), in the presence (+) or absence (-) of 500 µM CuSO4, for 8 hours. The cultures were then normalized to the same cell concentration, and serial 5-fold dilutions of each were spotted onto a YPEG agar plate and grown for 24 hours at 30°C.

Figure S2

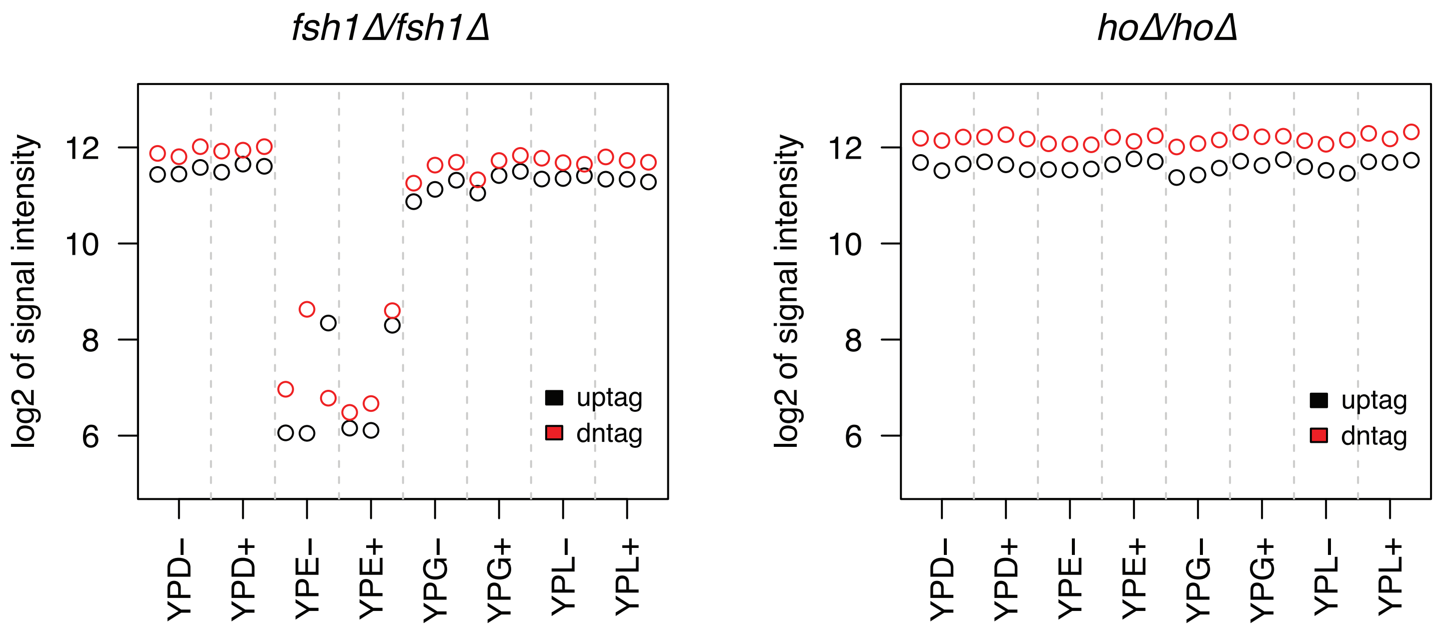

fsh1Δ/fsh1Δ
hoΔ/hoΔ
log2 of signal intensity
12
10
8
6
uptag
dntag
YPD−
YPD+
YPE−
YPE+
YPG−
YPG+
YPL−
YPL+

Figure S2. The FSH1 gene is essential for growth in media containing ethanol as the sole carbon source, but not in media containing glycerol or lactate as the sole carbon source. Dotplots illustrating the microarray log2-transformed fluorescence values (y-axis) for barcodes derived from the fsh1Δ/fsh1Δ strain (left plot) and hoΔ/hoΔ control strain (right plot), following competitive growth in the homozygous deletion pool. Both strains contain two unique barcodes; 'uptags' are indicated in black, and 'downtags' are indicated in red. The pool was grown under the following conditions (arranged on the x-axis): YP media plus dextrose (YPD), ethanol (YPE), glycerol (YPG), or lactate (YPL), in the presence (+) or absence (-) of 500 µM $CuSO_4$.

Figure S3

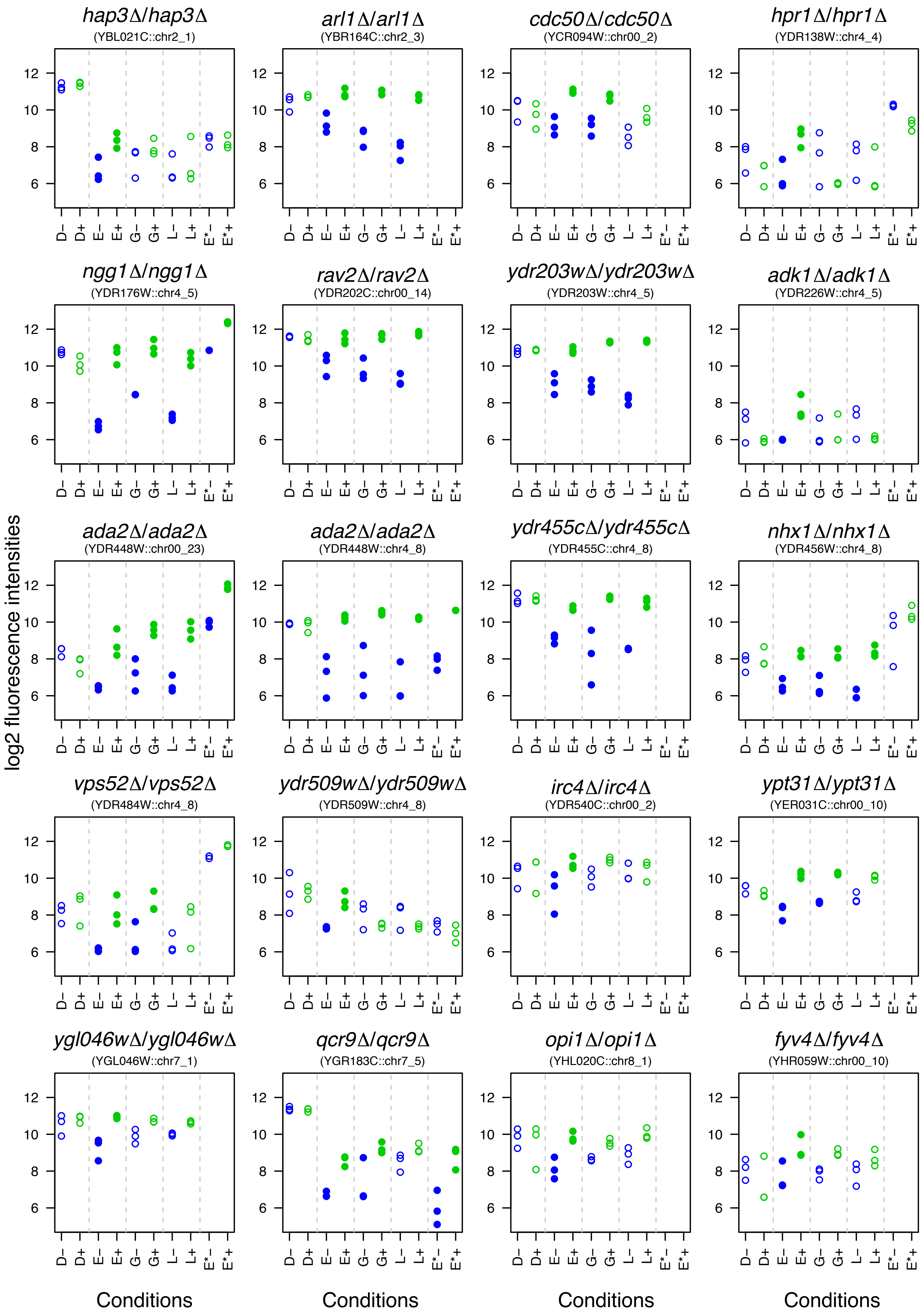
hap3Δ/hap3Δ
(YBL021C::chr2_1)
arl1Δ/arl1Δ
(YBR164C::chr2_3)
cdc50Δ/cdc50Δ
(YCR094W::chr00_2)
hpr1Δ/hpr1Δ
(YDR138W::chr4_4)
ngg1Δ/ngg1Δ
(YDR176W::chr4_5)
rav2Δ/rav2Δ
(YDR202C::chr00_14)
ydr203wΔ/ydr203wΔ
(YDR203W::chr4_5)
adk1Δ/adk1Δ
(YDR226W::chr4_5)
ada2Δ/ada2Δ
(YDR448W::chr00_23)
ada2Δ/ada2Δ
(YDR448W::chr4_8)
ydr455cΔ/ydr455cΔ
(YDR455C::chr4_8)
nhx1Δ/nhx1Δ
(YDR456W::chr4_8)
vps52Δ/vps52Δ
(YDR484W::chr4_8)
ydr509wΔ/ydr509wΔ
(YDR509W::chr4_8)
irc4Δ/irc4Δ
(YDR540C::chr00_2)
ypt31Δ/ypt31Δ
(YER031C::chr00_10)
ygl046wΔ/ygl046wΔ
(YGL046W::chr7_1)
qcr9Δ/qcr9Δ
(YGR183C::chr7_5)
opi1Δ/opi1Δ
(YHL020C::chr8_1)
fyv4Δ/fyv4Δ
(YHR059W::chr00_10)
log2 fluorescence intensities
6
8
10
12
D− D+ E− E+ G− G+ L− L+ E*− E*+
Conditions

Figure S3 (continued)

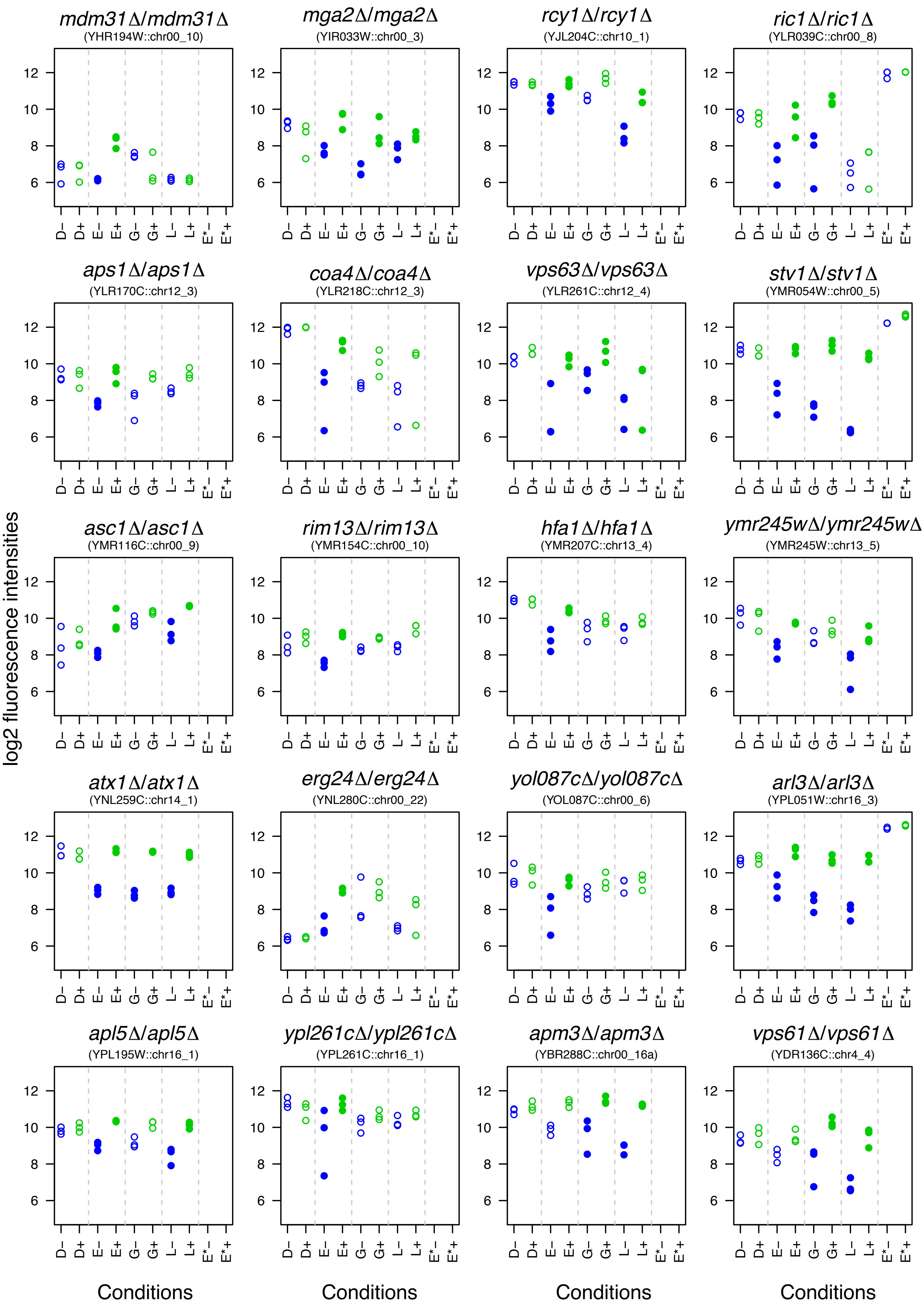

Figure S3 (continued)

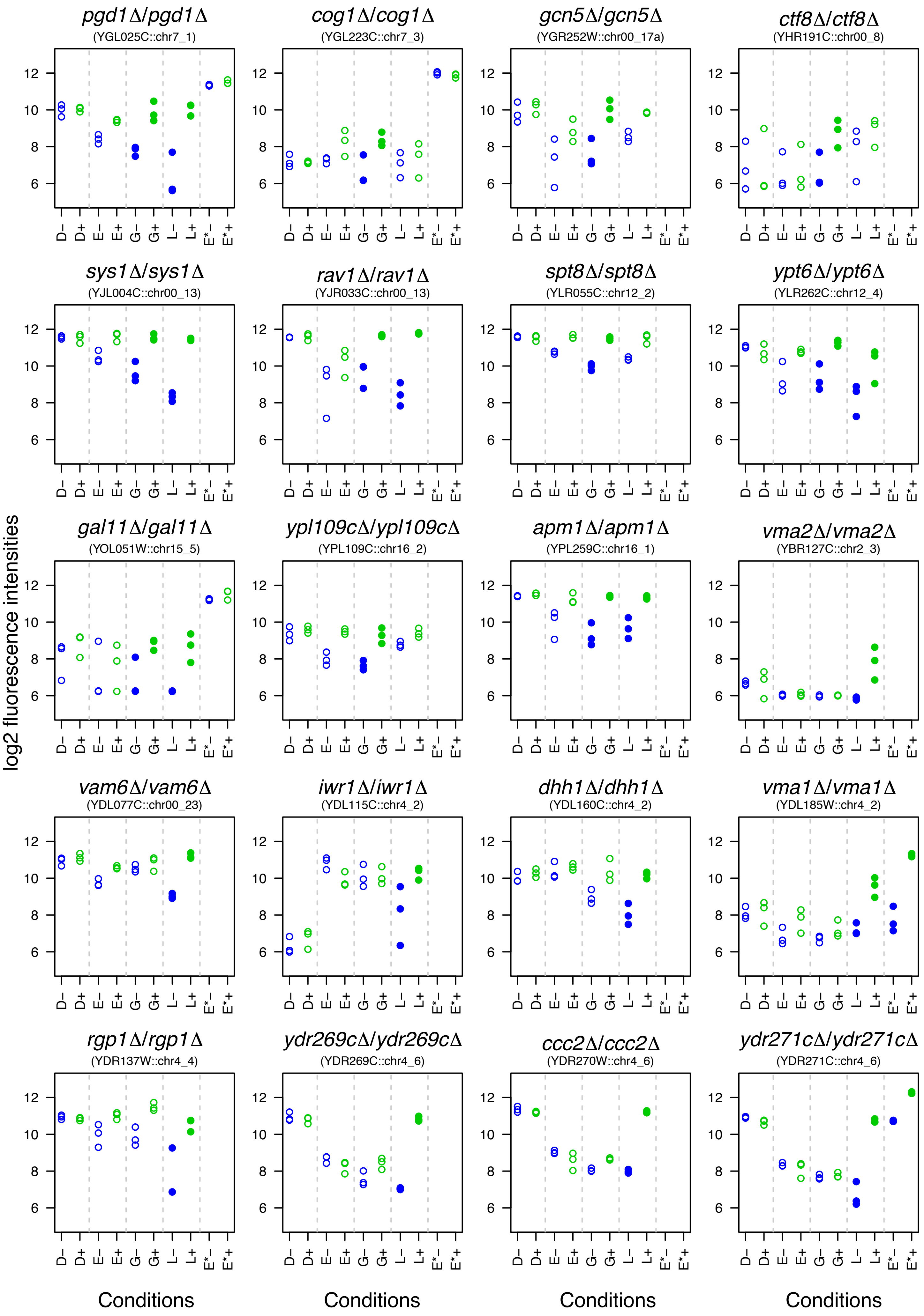

Figure S3 (continued)

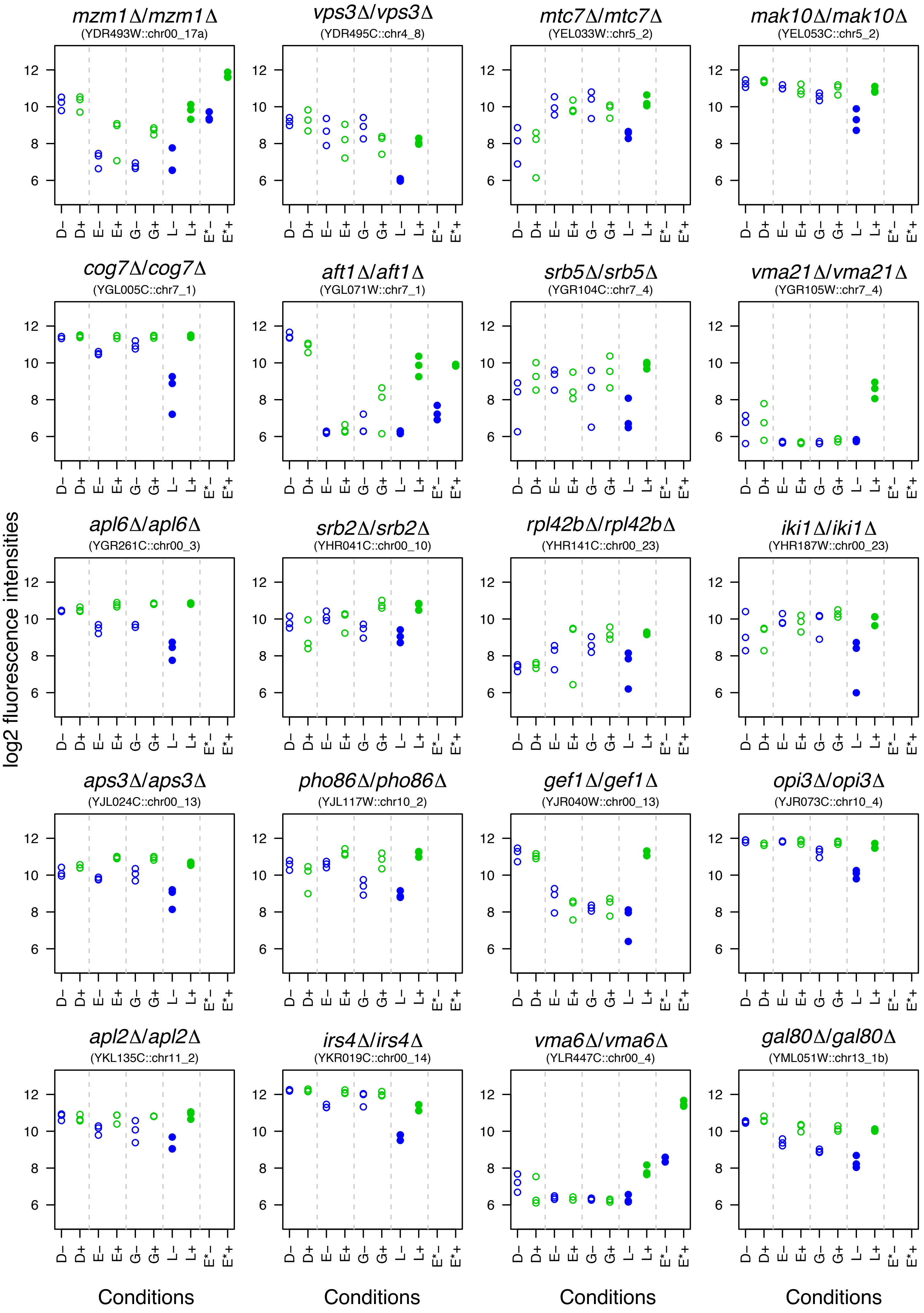

Figure S3 (continued)

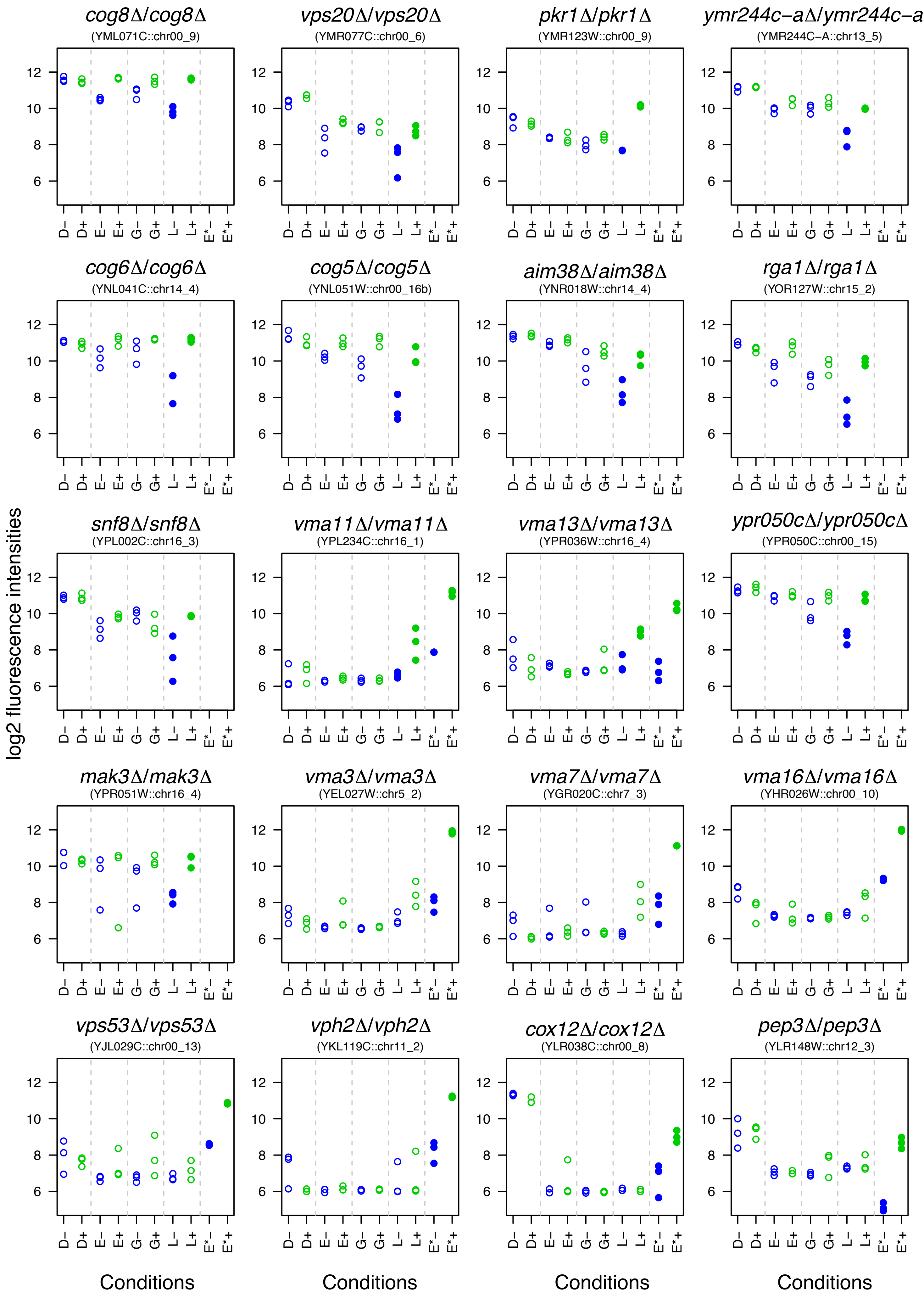

Figure S3 (continued)

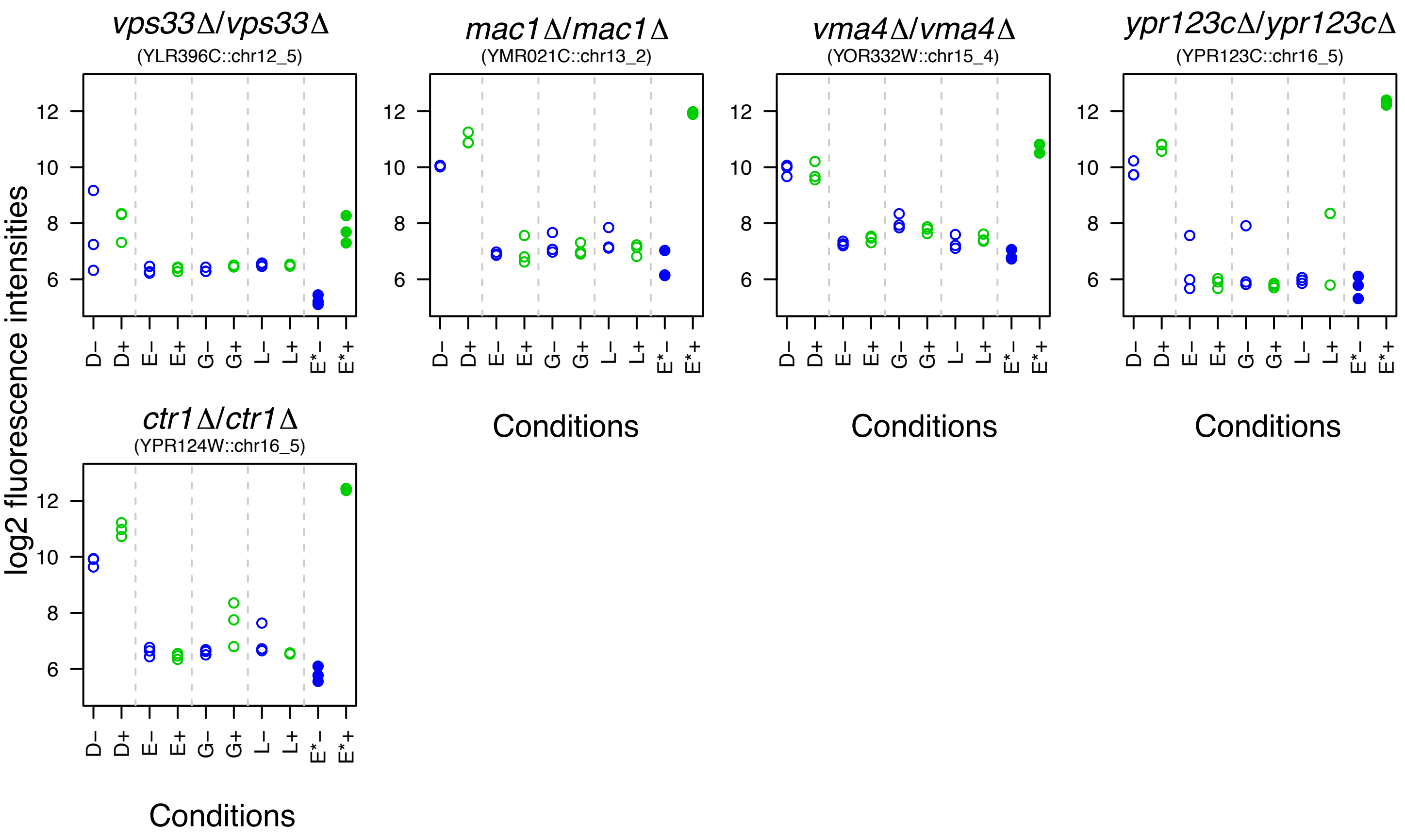

Figure S3. Deletion pool results for 105 strains in which the beneficial effects of $CuSO_4$ were greater compared to other strains in the pool. Each dotplot shows the quantile-normalized, log2-transformed fluorescence values (y-axis) of barcodes associated with the strain indicated above each plot. Most strains contain two unique barcodes, however only results for the barcode exhibiting the greatest difference between the $+CuSO_4$ and $-CuSO_4$ conditions are shown. Pool growth conditions are indicated on the x-axis. The complete homozygous deletion pool (n=5050 strains) was grown in YP (Yeast extract, Peptone) media plus dextrose (D), ethanol (E), glycerol (G), or lactate (L), whereas the pool of respiration deficient strains (n=331) was grown only in YP media plus ethanol (E*). Data derived from growth in the presence (+) or absence (-) of 500 µM $CuSO_4$ are colored in green and blue, respectively. Data from triplicate experiments are shown, and are represented by filled circles if the difference between the mean of the $+CuSO_4$ replicates and $-CuSO_4$ replicates is significant (q-value < 0.05) and greater than 1.5. This was the criterion used to identify these 105 strains.

Figure S4

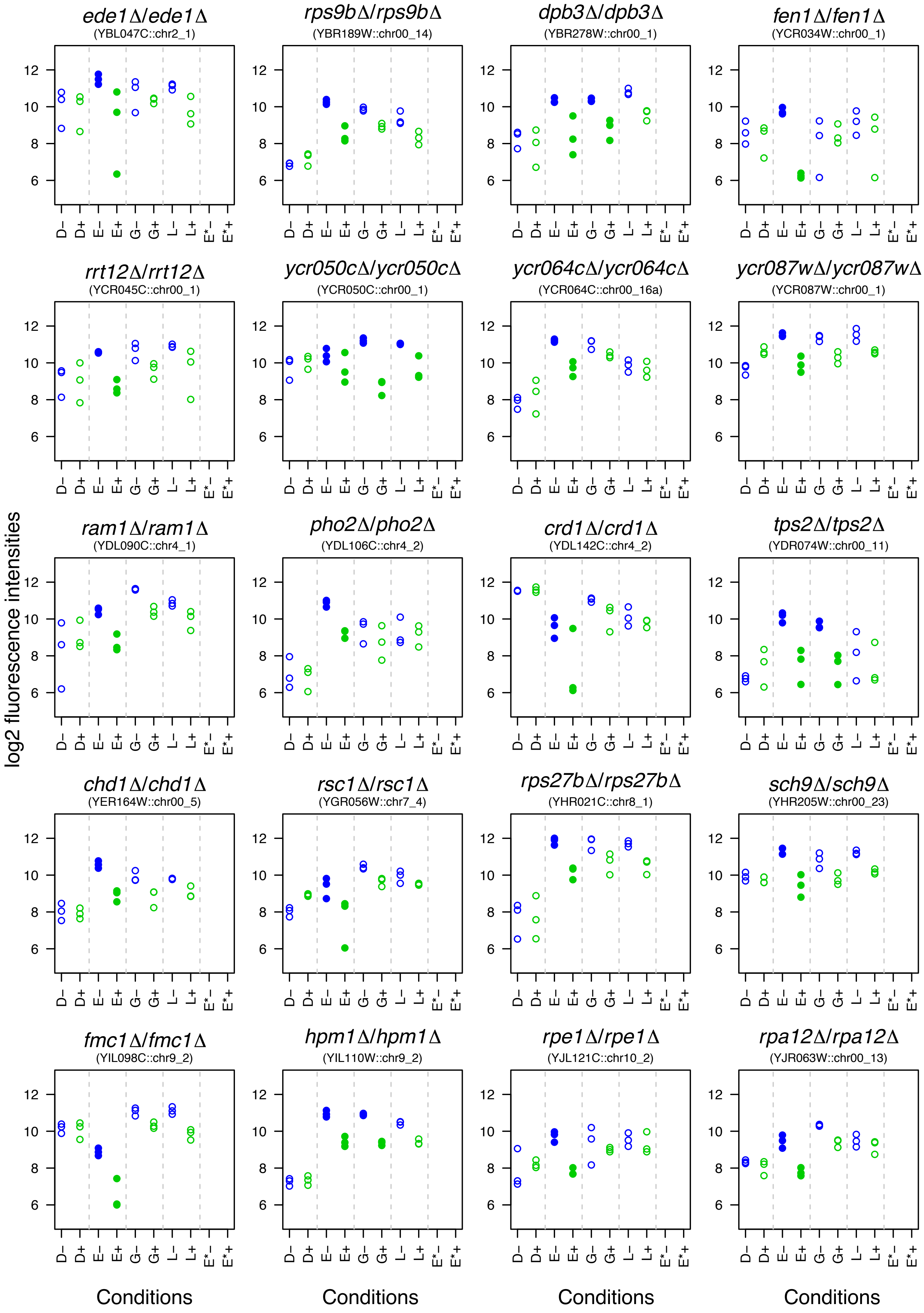

ede1Δ/ede1Δ
(YBL047C::chr2_1)
rps9bΔ/rps9bΔ
(YBR189W::chr00_14)
dpb3Δ/dpb3Δ
(YBR278W::chr00_1)
fen1Δ/fen1Δ
(YCR034W::chr00_1)
rrt12Δ/rrt12Δ
(YCR045C::chr00_1)
ycr050cΔ/ycr050cΔ
(YCR050C::chr00_1)
ycr064cΔ/ycr064cΔ
(YCR064C::chr00_16a)
ycr087wΔ/ycr087wΔ
(YCR087W::chr00_1)
ram1Δ/ram1Δ
(YDL090C::chr4_1)
pho2Δ/pho2Δ
(YDL106C::chr4_2)
crd1Δ/crd1Δ
(YDL142C::chr4_2)
tps2Δ/tps2Δ
(YDR074W::chr00_11)
chd1Δ/chd1Δ
(YER164W::chr00_5)
rsc1Δ/rsc1Δ
(YGR056W::chr7_4)
rps27bΔ/rps27bΔ
(YHR021C::chr8_1)
sch9Δ/sch9Δ
(YHR205W::chr00_23)
fmc1Δ/fmc1Δ
(YIL098C::chr9_2)
hpm1Δ/hpm1Δ
(YIL110W::chr9_2)
rpe1Δ/rpe1Δ
(YJL121C::chr10_2)
rpa12Δ/rpa12Δ
(YJR063W::chr00_13)
log2 fluorescence intensities
6
8
10
12
D– D+ E– E+ G– G+ L– L+ E*– E*+
Conditions

Figure S4 (continued)

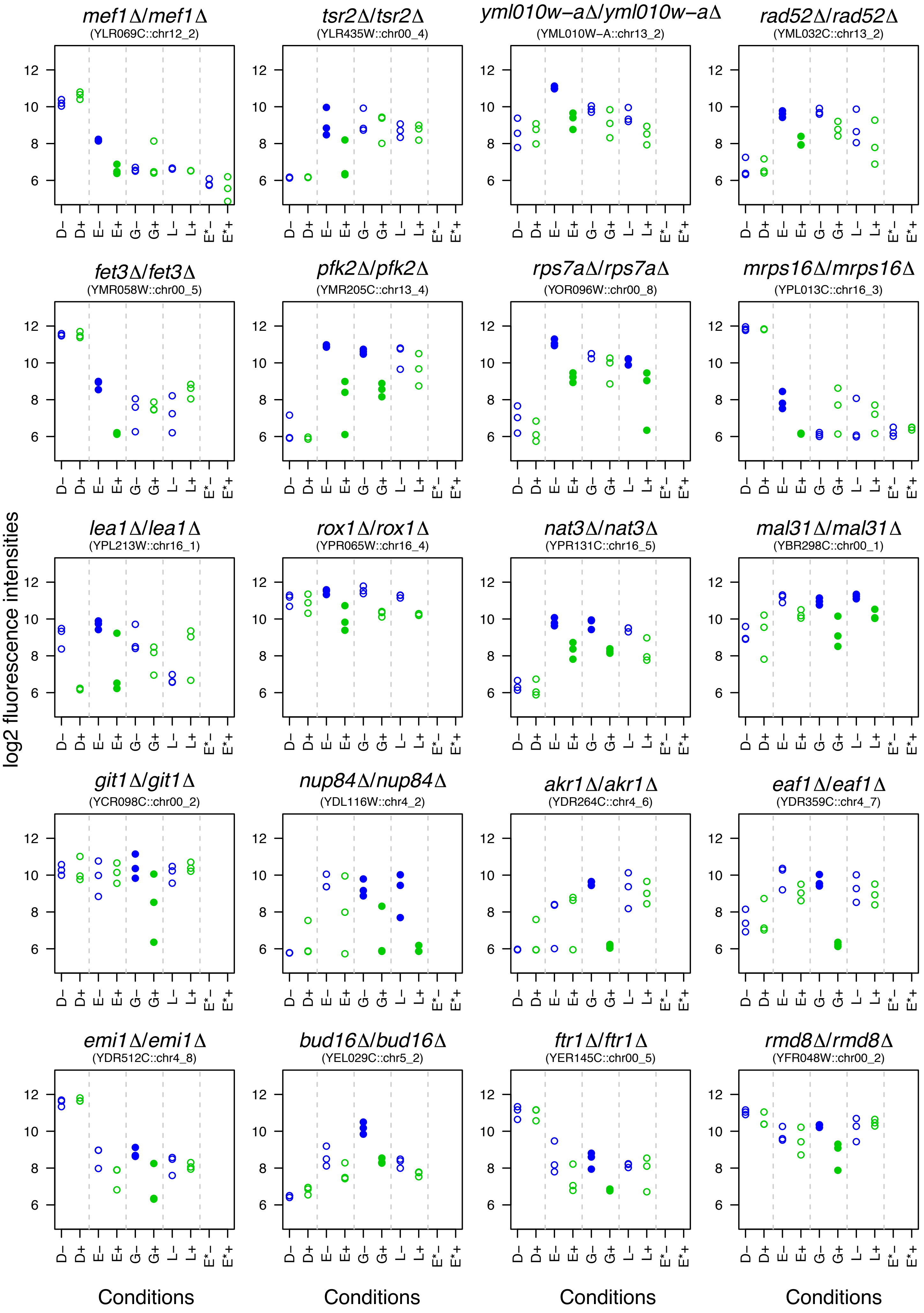

Figure S4 (continued)

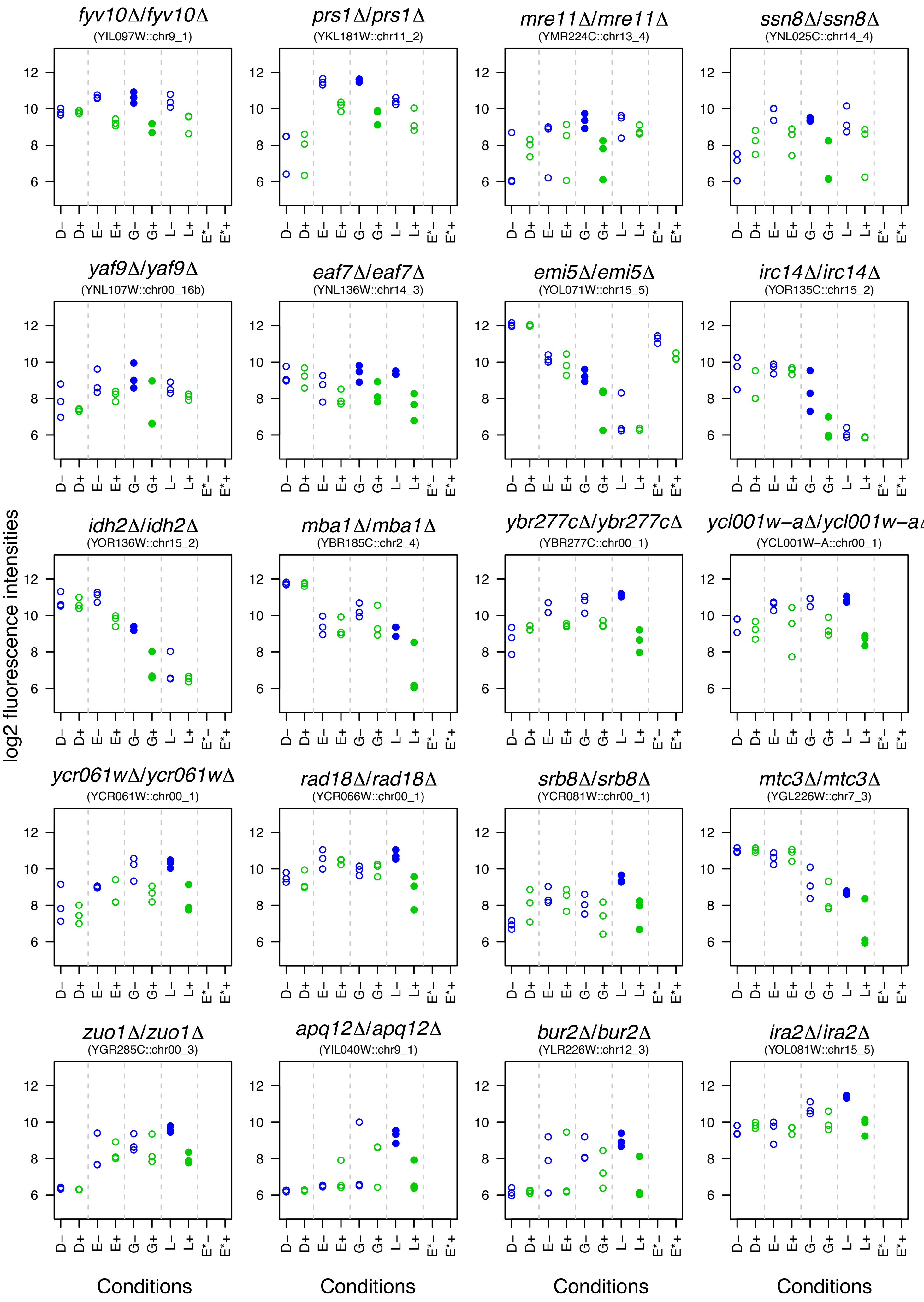

Figure S4 (continued)

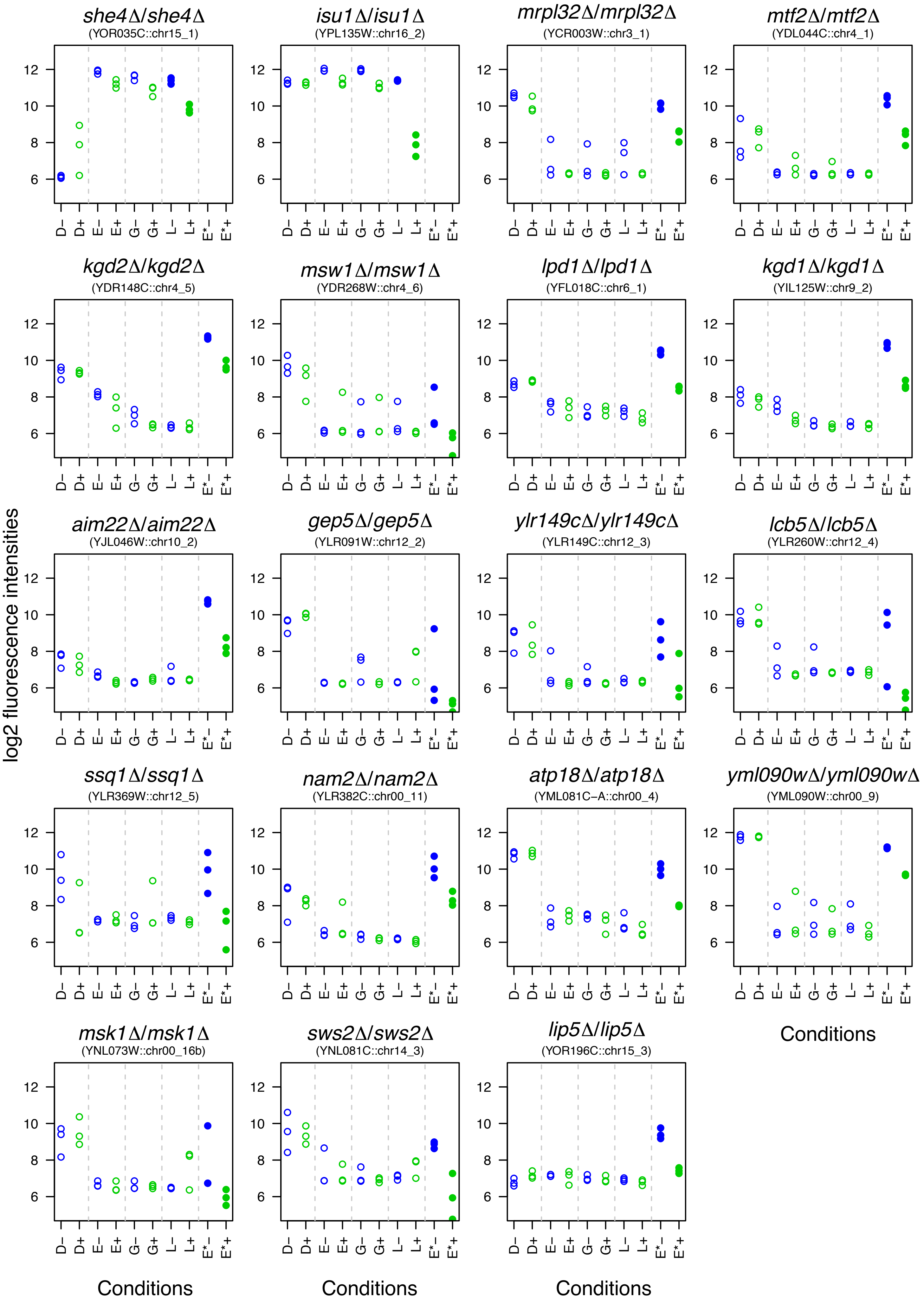

Figure S4. Deletion pool results for 79 strains in which the beneficial effects of $CuSO_4$ were smaller than other strains in the pool. Each dotplot shows the quantile-normalized, log2-transformed fluorescence values (y-axis) of barcodes associated with the strain indicated above each plot. Most strains contain two unique barcodes, however only results for the barcode exhibiting the greatest difference between the +$CuSO_4$ and -$CuSO_4$ conditions are shown. Pool growth conditions are indicated on the x-axis. The complete homozygous deletion pool (n=5050 strains) was grown in YP (Yeast extract, Peptone) media plus dextrose (D), ethanol (E), glycerol (G), or lactate (L), whereas the pool of respiration deficient strains (n=331) was grown only in YP media plus ethanol (E*). Data derived from growth in the presence (+) or absence (-) of 500 µM $CuSO_4$ are colored in green and blue, respectively. Data from triplicate experiments are shown, and are represented by filled circles if the difference between the mean of the -$CuSO_4$ replicates and that of the +$CuSO_4$ replicates is significant (q-value < 0.05) and smaller than -1.5. This was the criterion used to identify these 79 strains.

Figure S5

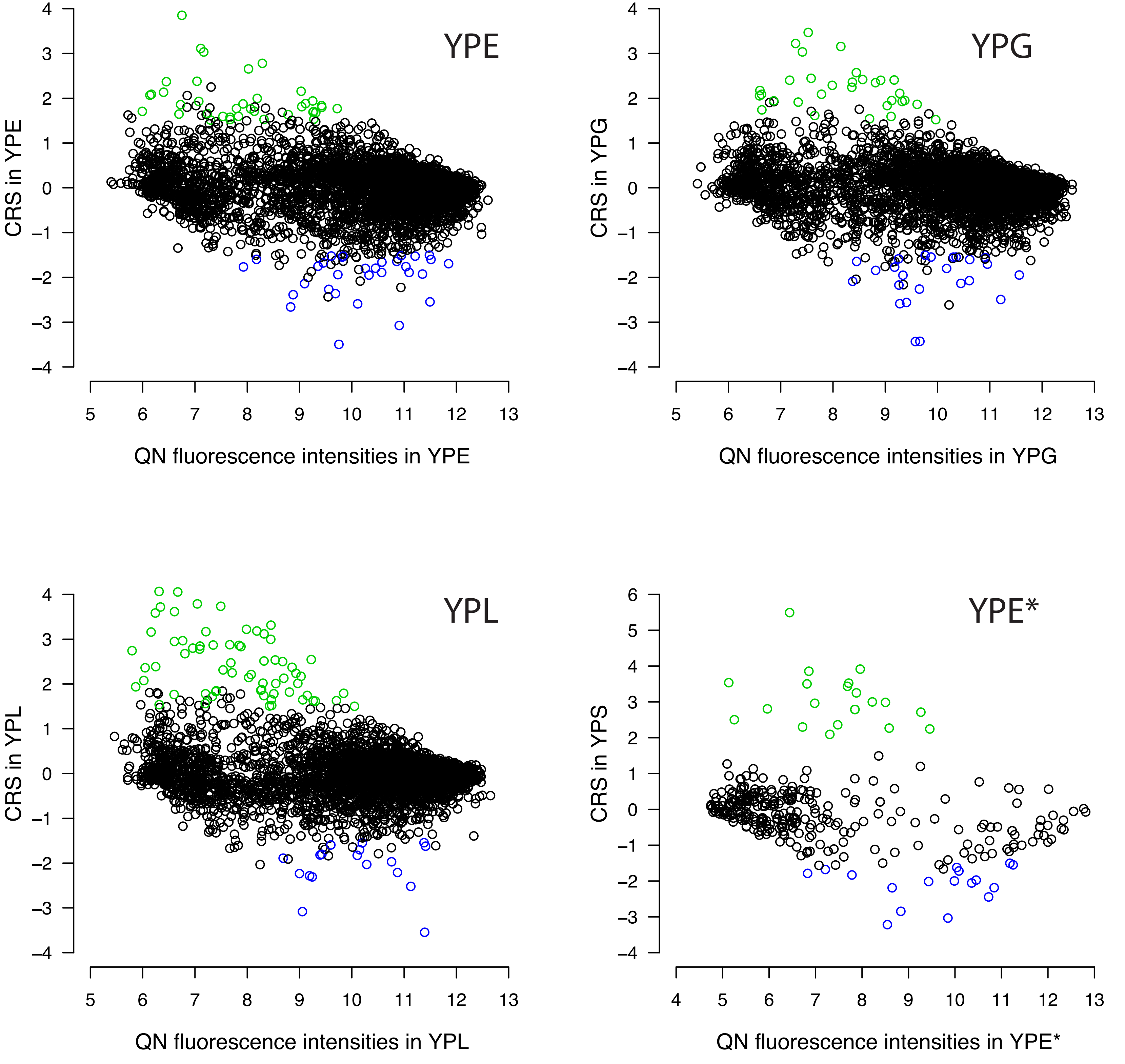
YPE
CRS in YPE
QN fluorescence intensities in YPE
YPG
CRS in YPG
QN fluorescence intensities in YPG
YPL
CRS in YPL
QN fluorescence intensities in YPL
YPE*
CRS in YPS
QN fluorescence intensities in YPE*

Figure S5. Scatterplots depicting the relationship between Copper Response Score (CRS, plotted on the y-axis) and respiratory fitness. Respiratory fitness is represented by the mean (of three replicates) quantile-normalized (QN) fluorescence signal of tags following competitive growth (plotted on the x-axis). The four plots show data for each strain in each of the four respiratory pool assays; i.e. the homozygous diploid (HD) deletion pool in ethanol-, glycerol-, or lactate-containing media (YPE, YPG, and YPL), and the respiratory-deficient (RD) deletion pool in ethanol-containing medium (YPE*). Only the fluorescence values of the tag used to calculate CRS are plotted (see Materials and Methods). Strains exhibiting a reduced Cu-boost (i.e. CRS smaller than -1.5 and a q-value < 0.05) are colored blue. Strains exhibiting an increased Cu-boost (i.e. CRS greater than 1.5 and a q-value < 0.05) are colored green and tended to have lower QN fluorescence signal (i.e. a respiratory growth defect).

Figure S6

A

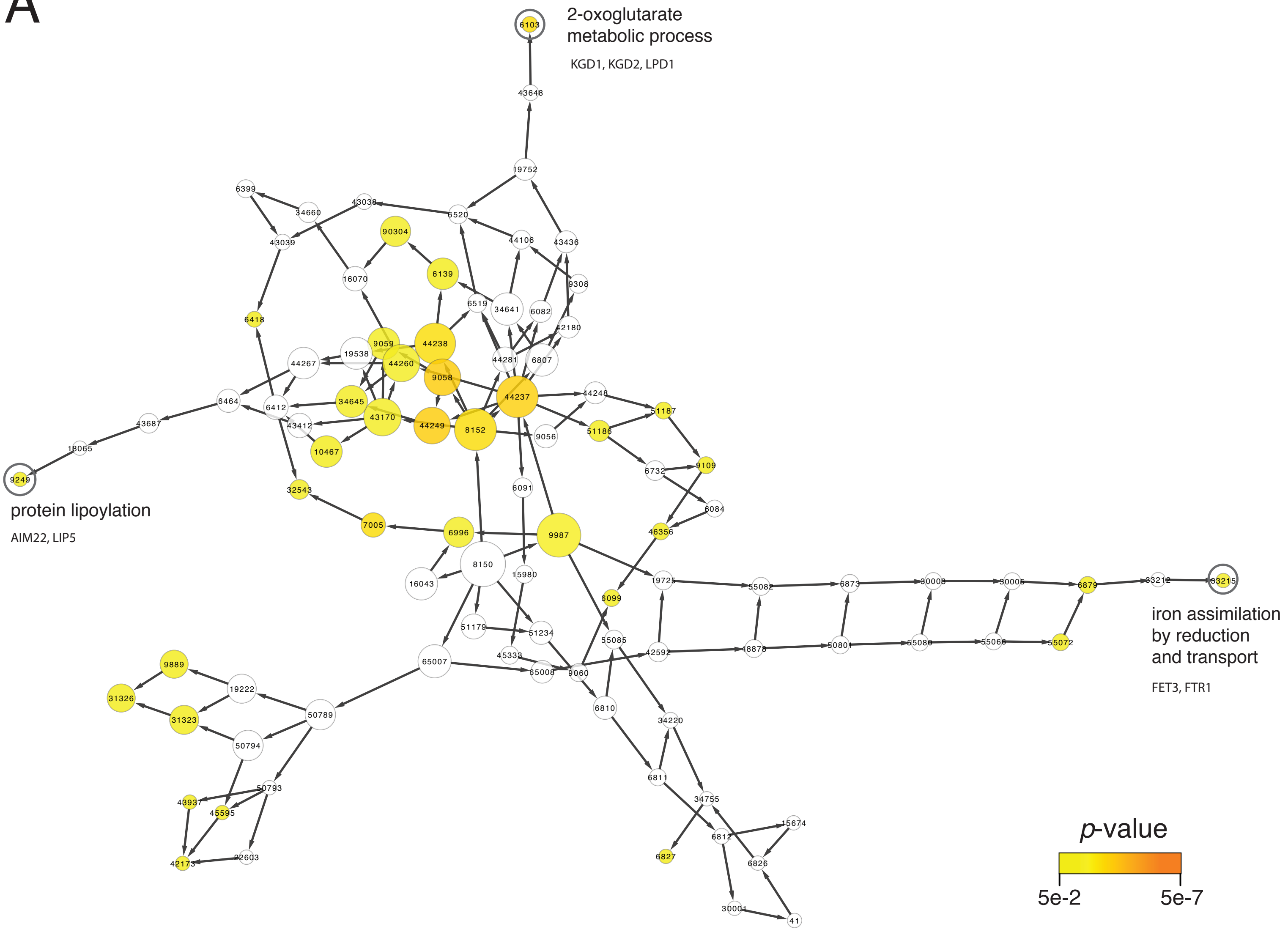
2-oxoglutarate metabolic process
KGD1, KGD2, LPD1
protein lipoylation
AIM22, LIP5
iron assimilation by reduction and transport
FET3, FTR1
p-value
5e-2
5e-7

Figure S6

B

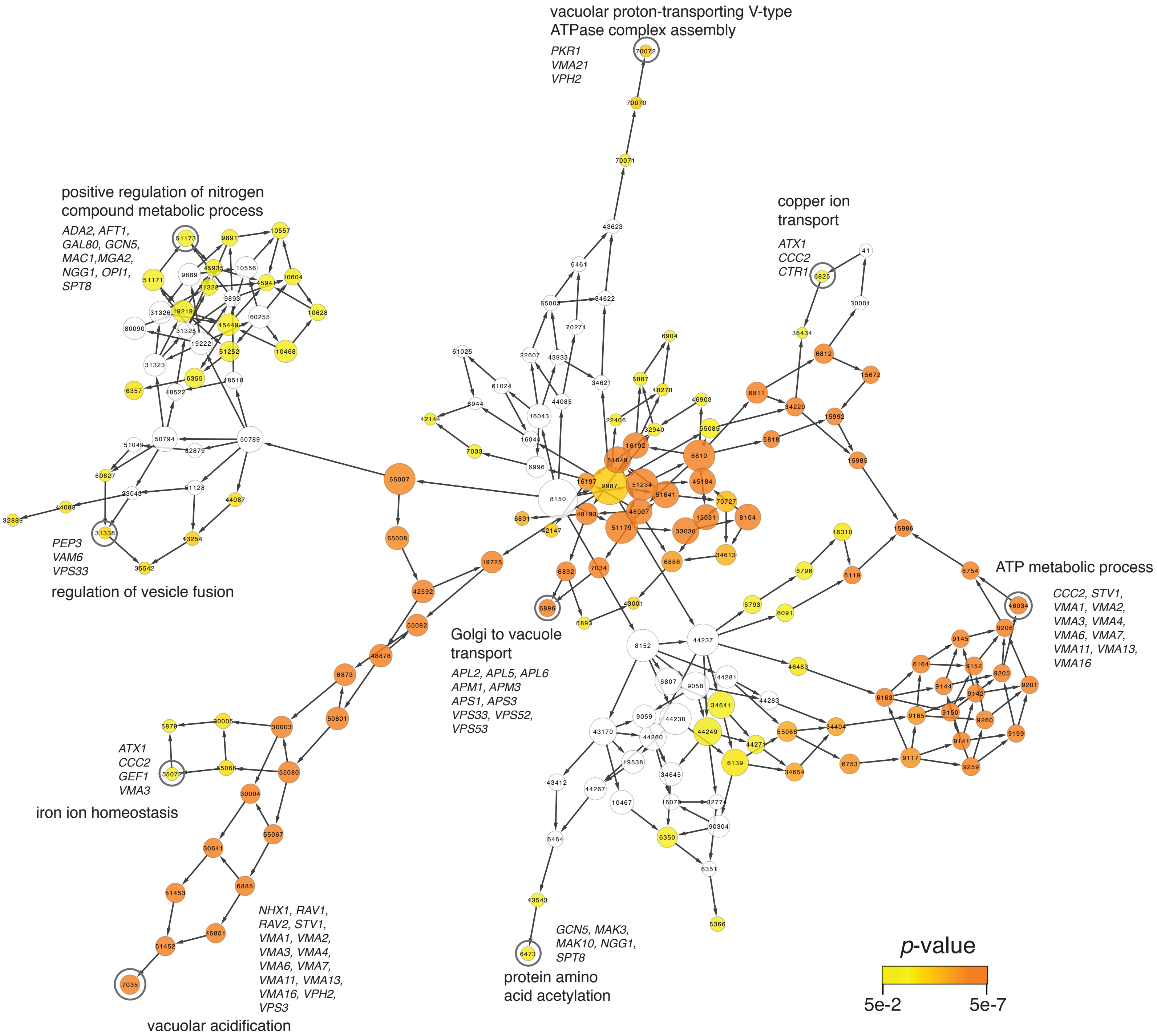
vacuolar proton-transporting V-type ATPase complex assembly
PKR1
VMA21
VPH2
positive regulation of nitrogen compound metabolic process
ADA2, AFT1, GAL80, GCN5, MAC1,MGA2, NGG1, OPI1, SPT8
copper ion transport
ATX1
CCC2
CTR1
PEP3
VAM6
VPS33
regulation of vesicle fusion
ATP metabolic process
CCC2, STV1, VMA1, VMA2, VMA3, VMA4, VMA6, VMA7, VMA11, VMA13, VMA16
Golgi to vacuole transport
APL2, APL5, APL6
APM1, APM3
APS1, APS3
VPS33, VPS52,
VPS53
ATX1
CCC2
GEF1
VMA3
iron ion homeostasis
NHX1, RAV1, RAV2, STV1, VMA1, VMA2, VMA3, VMA4, VMA6, VMA7, VMA11, VMA13, VMA16, VPH2, VPS3
vacuolar acidification
GCN5, MAK3, MAK10, NGG1, SPT8
protein amino acid acetylation
p-value
5e-2
5e-7

Figure S6. Network representation of Gene Ontology (GO) categories that are significantly over-represented among the 79 strains exhibiting diminished Cu-dependent growth (A) and 105 strains exhibiting enhanced Cu-dependent growth (B). Nodes are labeled with their respective Gene Ontology identifiers, which are also listed in Tables S2 and S3. Representative nodes are circled and labeled with those genes identified in the screen. The BiNGO plugin for Cytoscape was used to analyze the gene lists, and to generate the network images.

Figure S7

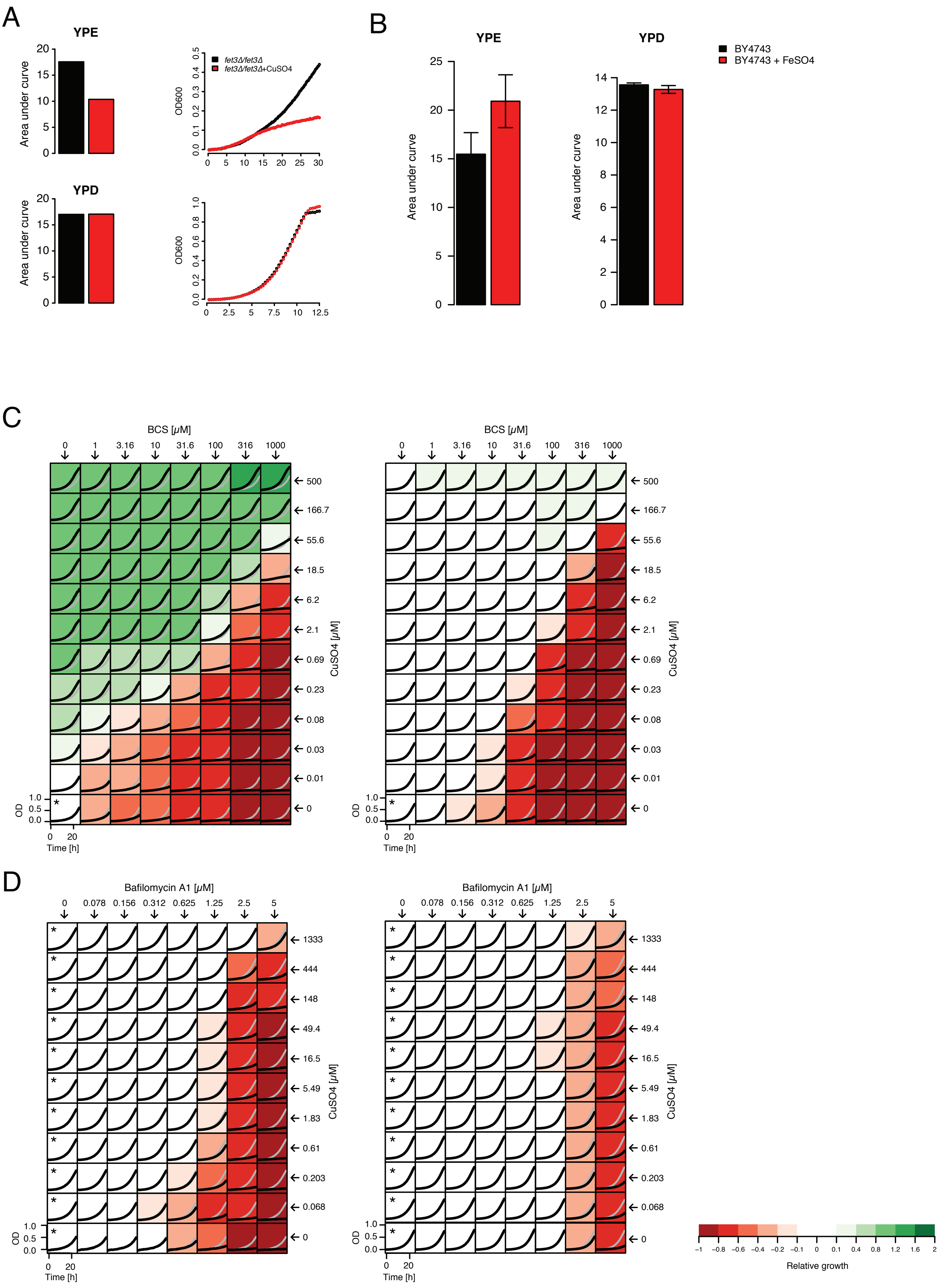
A
YPE
YPD
Area under curve
OD600
fet3Δ/fet3Δ
fet3Δ/fet3Δ+CuSO4
B
BY4743
BY4743 + FeSO4
C
BCS [μM]
CuSO4 [μM]
OD
Time [h]
D
Bafilomycin A1 [μM]
CuSO4 [μM]
Relative growth

Figure S7. Excess $FeSO_4$ masks the effects of $CuSO_4$ on respiratory growth. (A) Respiratory and fermentative growth of the *fet3Δ/fet3Δ* deletion strain in the presence or absence of 500 µM $CuSO_4$ (see legend). Growth after 30 and 12.5 hours in YPE (top) and YPD (bottom), respectively, was measured by area under the curve (AUC; y-axis of the barplots). The corresponding growth curves are shown on the right of each barplot. (B) Respiratory and fermentative growth of BY4743 in the presence or absence of 1 mM $FeSO_4$ (see legend). Growth after 25 and 12.5 hours in YPE (top) and YPD (bottom), respectively, was measured by area under the curve (AUC; y-axis of the barplots). The mean of three replicates is plotted; error bars represent the standard deviation. (C) Dose-response matrices comparing the combinatorial effects of $CuSO_4$ and BCS on respiratory growth in the absence (left panel) and presence (right panel) of 1 mM $FeSO_4$. The left panel is identical to Figure 1A and is included here for reference. Yeast (BY4743) was grown under respiring conditions (YPE media) in increasing concentrations of $CuSO_4$ (see right) and BCS (see top). Optical density (*i.e.* OD_{600}, y-axis) of each culture is plotted over time (x-axis). Each condition is colored based on growth relative to the untreated condition (*i.e.* no BCS and no $CuSO_4$ added; marked with an asterisk and represented by the grey curve). Green and red indicate increased and decreased growth relative to this reference, respectively (see legend). (D) Dose-response matrices comparing the combinatorial effects of $CuSO_4$ and bafilomycin A1 on respiratory growth in the absence (left panel) and presence (right panel) of 1 mM $FeSO_4$. The left panel is identical to Figure 3C and is included here for reference. BY4743 was grown in increasing concentrations of $CuSO_4$ (see right) and bafilomycin A1 (see top). Optical density (*i.e.* OD_{600}, y-axis) of each culture is plotted over time (x-axis). Color-coding is identical to (C). Asterisks and grey curves indicate the reference in every row.

Figure S8

A

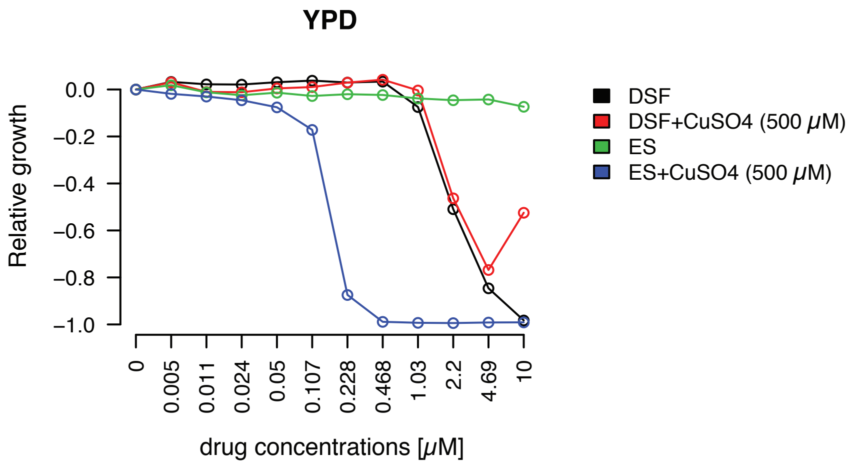

B

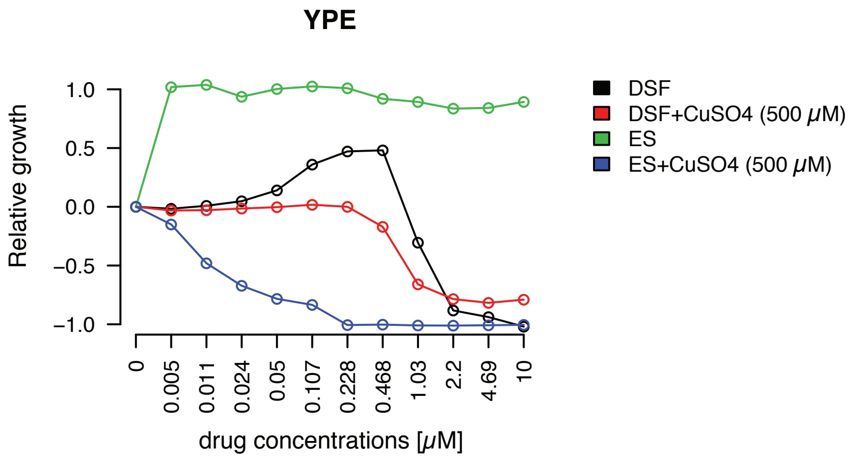

C

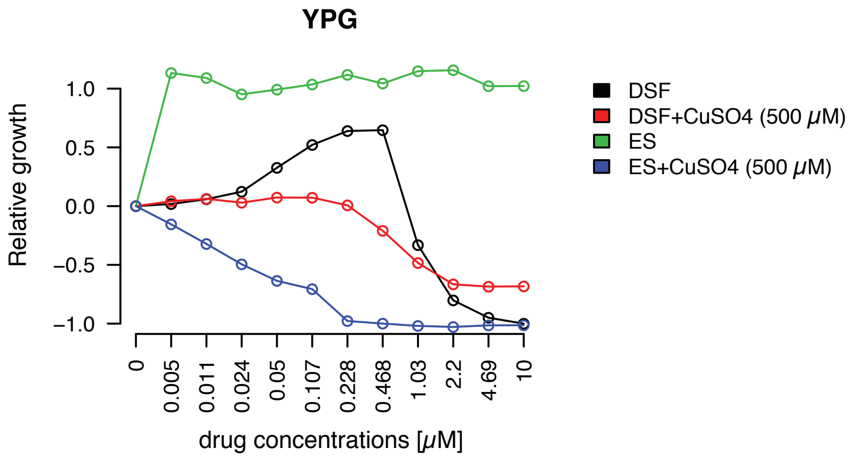

Figure S8. Elesclomol (ES) exhibits greater copper-dependent toxicity than disulfiram (DSF). Dose-dependent effects of ES and DSF on growth of wild-type yeast (BY4743) in the presence or absence of 500 μM $CuSO_4$ (see legend). Growth relative to that in the absence of drug is indicated by the y-axis, and is plotted for each concentration of drug tested (indicated on the x-axis). Results for growth in YPD (A), YPE (B), and YPG (C) are presented.

Figure S9

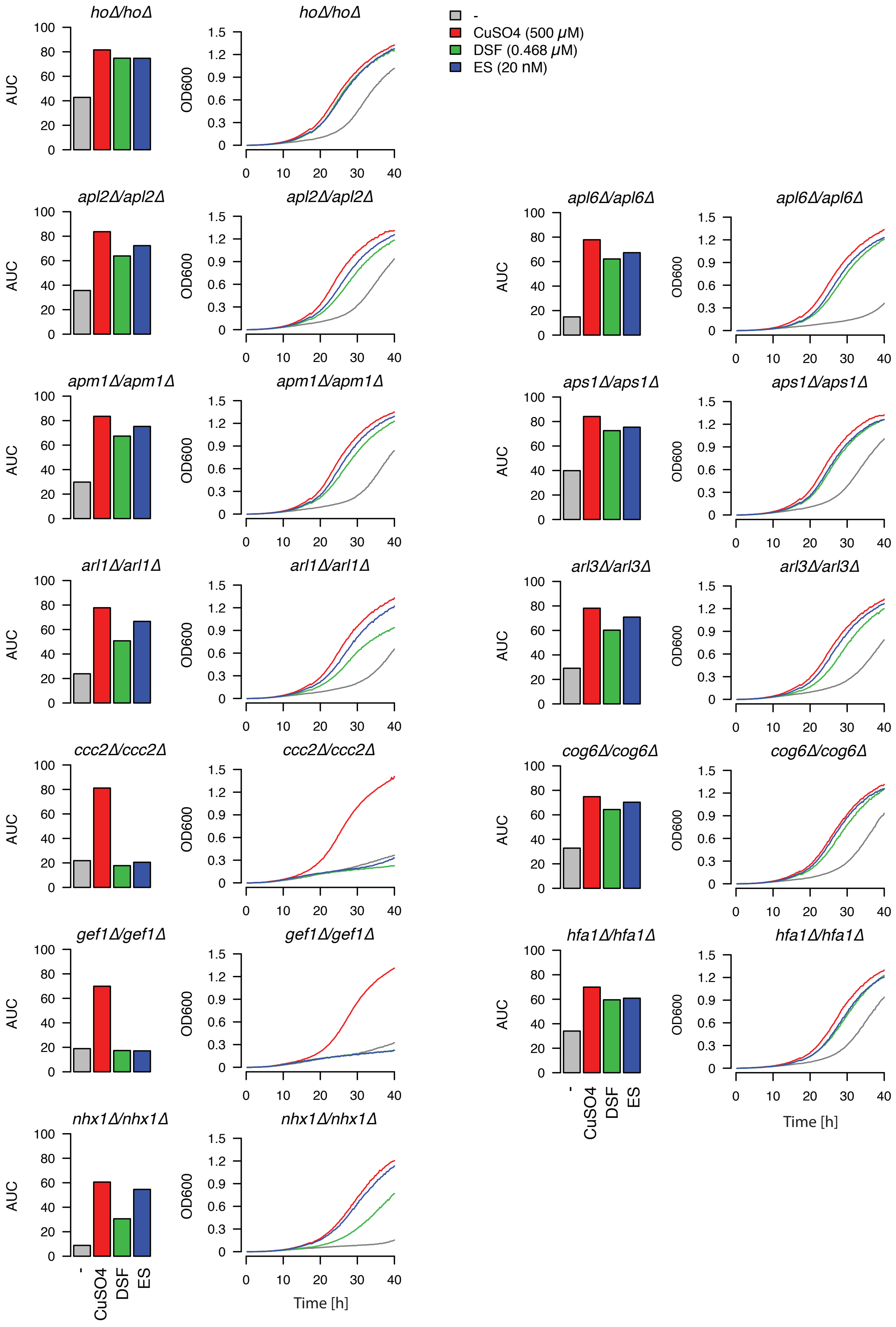

-
CuSO4 (500 μM)
DSF (0.468 μM)
ES (20 nM)
hoΔ/hoΔ
apl2Δ/apl2Δ
apl6Δ/apl6Δ
apm1Δ/apm1Δ
aps1Δ/aps1Δ
arl1Δ/arl1Δ
arl3Δ/arl3Δ
ccc2Δ/ccc2Δ
cog6Δ/cog6Δ
gef1Δ/gef1Δ
hfa1Δ/hfa1Δ
nhx1Δ/nhx1Δ
AUC
OD600
0
20
40
60
80
100
0.3
0.6
0.9
1.2
1.5
10
30
40
-
CuSO4
DSF
ES
Time [h]

Figure S9. Rescue of Cu-deficiency phenotypes by $CuSO_4$, disulfiram (DSF), or elesclomol (ES). Respiratory growth of the *hoΔ/hoΔ* deletion strain (control) and 11 deletion strains listed in Table 1 in response to 500 µM $CuSO_4$ (red), 0.468 µM DSF (green), 20 nM ES (blue), or no treatment (gray). Growth after 40 hours in YPE was measured by area under the curve (AUC; y-axis of the barplots). The corresponding growth curves are shown on the right of each barplot.
